# Harnessing the thermotolerant methylotroph *Bacillus methanolicus* for methanol-based synthetic L-proline production

**DOI:** 10.1186/s12934-026-03032-8

**Published:** 2026-05-23

**Authors:** Christine Frank, David Virant, Gregor Kosec, Tamara Hoffmann, Oskar Zelder, Max F. Felle, Erhard Bremer

**Affiliations:** 1 Faculty of Biology, Microbiology, Marburg University, 35043 Marburg, Germany; 2 Acies Bio d.o.o, Tehnoloski Park 21, 1000 Ljubljana, Slovenia; 3 Center for Synthetic Microbiology (SYNMIKRO), Marburg University, Karl-Von-Frisch Strasse 14, 35043 Marburg, Germany; 4 BASF SE, RGR/D, A030, 67056 Ludwigshafen Am Rhein, Germany

**Keywords:** Bacilli, Amino acids, Methanol, Synthetic microbiology, Cell factory, Excretion, High temperature

## Abstract

**Background:**

*Bacillus methanolicus* MGA3 is a thermotolerant methylotroph that utilizes methanol, a renewable C₁ substrate, as its sole carbon and energy source. The strain naturally overproduces and secretes L-glutamate, making it a promising platform for engineering pathways toward L-glutamate-derived amino acids such as L-proline, which has applications in nutrition, stress protection, and industry.

**Results:**

Heterologous expression of an osmotic stress–responsive L-proline biosynthetic operon from the mesophile *Bacillus licheniformis* in the thermotolerant *B. methanolicus* strain MGA3 did not increase L-proline levels but instead led to accumulation of L-citrulline. This was likely due to heat sensitivity of pyrroline-5-carboxylate reductase (ProH), the last enzyme of the osmoregulatory L-proline biosynthetic route, and metabolic crosstalk between L-proline and L-arginine pathways operating in Bacilli. To overcome these limitations, a synthetic operon containing the native anabolic *proBA* and *proI* L-proline biosynthetic genes from *B. methanolicus* MGA3 was engineered to remove transcriptional T-box regulation and biochemical feedback inhibition of ProB enzyme activity. Expression of this engineered operon enhanced L-proline synthesis and triggered its secretion during methanol-based growth of *B. methanolicus* MGA3 at 50° C. In fed-batch fermentation with methanol as carbon and energy source, extracellular L-proline levels reached 262 ± 20 mg L⁻^1^ after 40 h. During the fermentation process, a stepwise increase in medium osmolarity was observed, likely due to large-scale L-glutamate excretion, which impaired cellular growth.

**Conclusions:**

This study links osmolarity dynamics to methanol-based fermentation in *B. methanolicus* MGA3 and demonstrates its potential as a cell factory for L-proline and L-citrulline production. These findings support further strain optimization for producing value-added amino acids and highlight the relevance of methylotrophic thermophiles in sustainable biotechnology.

**Supplementary Information:**

The online version contains supplementary material available at 10.1186/s12934-026-03032-8.

## Background

Natural and engineered microbial cell factories are well-established workhorses in biotechnology, particularly for the large-scale production of amino acids, compounds with considerable commercial value [1–3]. As these advanced bioprocesses typically rely on sugar- or glycerol-based feedstocks, there is a growing interest in shifting to alternative carbon sources that minimize competition with human and animal nutrition and help lower the carbon footprint of industrial fermentation processes [4]. Renewable methanol has emerged as a promising candidate in this context [5, 6]. As a highly reduced, energy-rich C_1_-compound, methanol can be sustainably produced from biomass or captured from industrial CO₂ emissions. It is fully miscible with water and can be completely metabolized by methylotrophic microorganisms during fermentation [7, 8]. These favorable properties have sparked significant interest in the development of robust synthetic methylotrophic microbial chassis strains for eco-friendly methanol-based bioproduction [9–14], while also renewing attention to natural microbial methylotrophs such as *Bacillus methanolicus* [15, 16] as sustainable platforms for industrial biotechnology [17–19].

The *B. methanolicus* strain MGA3 has attracted attention because it can metabolize not only methanol but also alternative substrates such as mannitol, arabitol, and seaweed extracts at high temperature [17, 18, 20, 21]. Such metabolic versatility [22, 23] facilitates growth and genetic manipulation of *B. methanolicus* MGA3 under laboratory conditions [24–27] and enables the use of readily available alternative substrates for fermentation processes. Originally isolated from freshwater marsh soil, this bacterium can grow in seawater and exhibits optimal growth at elevated temperatures of approximately 50° C [15, 16, 28]. Among the few available isolates of this species, strain MGA3 stands out as a natural overproducer of L-glutamate, which it efficiently secretes in very large quantities into the culture medium during methanol-based fermentation [23, 29]. The thermotolerant nature of *B. methanolicus* MGA3 offers several advantages for industrial biotechnology, including enhanced chemical and enzymatic reaction rates, reduced bioreactor cooling requirements, and a lower risk of microbial contamination during fermentation [19, 30–32]. Moreover, the natural overproduction of L-glutamate by *B. methanolicus* MGA3, a key metabolite at the intersection of carbon and nitrogen metabolism in many Bacilli [33], positions this strain as a promising chassis for sustainable, low-carbon biomanufacturing applications [17, 18, 23–27, 34, 35].

The successful use of *B. methanolicus* MGA3 for the heterologous production of biotechnologically relevant proteins and various bulk and fine chemicals, has already been demonstrated. Among the fine chemicals overproduced in *B. methanolicus* MGA3 and its mutant derivative are the amino acids L-glutamate and L-lysine [17, 23, 36], and in engineered strains, the production of L-arginine, riboflavin, acetoin, polyamines, cadaverine, aminovalerate, diaminobutyric acid (DABA) and C30 terpenoids was successfully achieved [20, 23, 25, 27, 37–41]. In parallel, ongoing efforts are dedicated to the development of molecular tools and genetic engineering strategies to enhance the manipulability and performance of *B. methanolicus* MGA3 as a robust and genetically accessible microbial cell factory [24–27, 32, 34, 37].

In the present study, we focus on extending this portfolio by conducting a *proof-of-concept* study for methanol-based production of L-proline. L-proline is the only cyclic proteogenic amino acid, establishing its unique role in protein folding and secondary structure [42, 43]. In microorganisms, L-proline serves multiple physiological functions: it acts as a nutrient, as an osmotic stress protectant, as a chemical chaperone, as a redox-balancing agent, as a source of energy via the Stickland reaction, and it has been implicated in bacterial virulence [42–47]. Although L-proline is biotechnologically not produced on the same industrial scale as the amino acids L-glutamate, L-lysine, or DL-methionine [1–3], there is growing interest in its biomanufacturing [48–54]. Current L-proline production volumes have reached several hundred metric tons per year and possess a estimated market value in 2023 of around 260 million USD (https://www.24chemicalresearch.com/reports/283877/global-regional-lproline-forecast-supply-dem-analysis-competitive-market-2025-2032-339). L-proline is used in various sectors, including cosmetics, medicine and the food industry, and holds potential as a feed additive. Moreover, it serves as a precursor for the synthesis of several hydroxy-L-proline isomers, valuable compounds with diverse applications in cosmetics, food products, and as chiral building blocks in the production of pharmaceutical ingredients and antibiotics [55, 56], product portfolios with a current estimated market value of about 80 million USD (https://www.databridgemarketresearch.com/reports/global-hydroxyproline-market?utm_source=chatgpt.com).

**Fig. 1:**
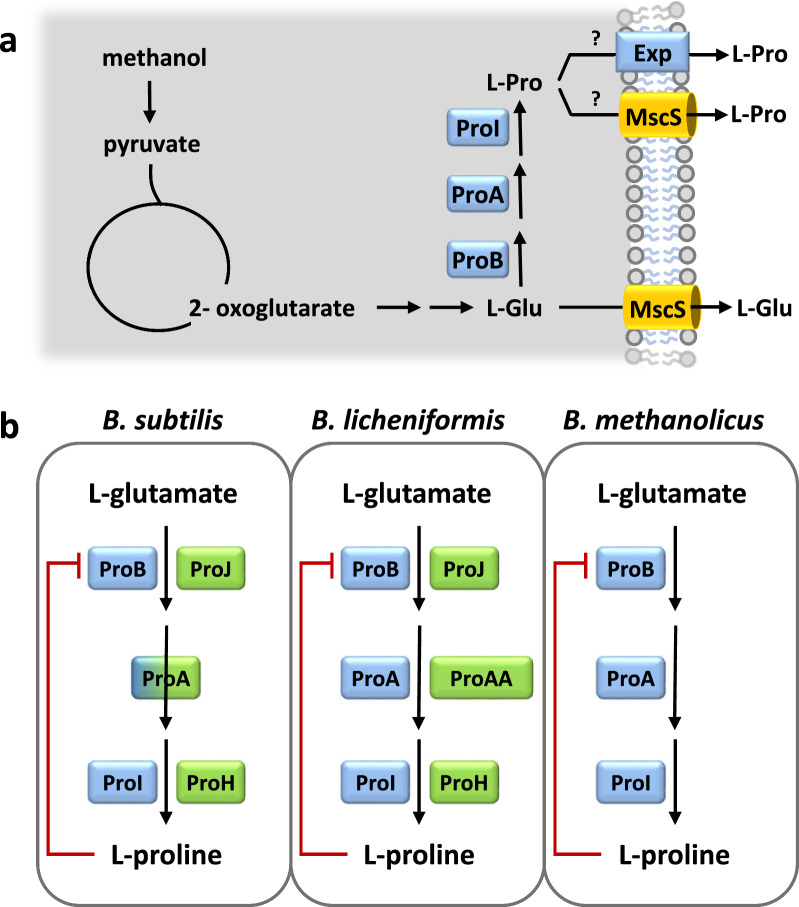
L-proline synthesis routes in *B. subtilis*, *B. licheniformis* and *B. methanolicus* MGA3. (**a**) Anabolic L-proline biosynthesis in *B. methanolicus* MGA3 proceeds from L-glutamate (L-Glu) to L-proline (L-Pro) via ProB (glutamate 5-kinase), ProA (γ-glutamyl phosphate reductase), and ProI (pyrroline-5-carboxylate reductase). L-glutamate export mainly occurs through an MscS-type mechanosensitive channel [29]; the precise nature of the L-proline exporter is unknown. (**b**) Comparison of anabolic and osmotic stress-responsive L-proline biosynthetic routes in *B. subtilis* JH642 [66], *B. licheniformis* DSM13 [64], and *B. methanolicus* MGA3 [16]. The anabolic pathway (ProB–ProA–ProI), feedback-inhibited at ProB, is shown in blue. Osmotic stress-responsive L-proline biosynthetic pathways (ProJ–ProA–ProH or ProJ–ProAA–ProH) are shown in green; ProJ is predicted to be insensitive to L-proline feedback inhibition [59]. In *B. subtilis*, both pathways converge at ProA [66, 68] whereas in *B. licheniformis* [64] they function independently

In a limited number of bacteria, L-proline can be synthesized from ornithine via ornithine cyclodeaminase (OCD; EC 4.3.1.12) [48]. However, in most bacteria capable of synthesizing L-proline, its biosynthetic route begins with the central metabolite L-glutamate (Fig. 1a, b) [42]. The L-glutamate to L-proline biosynthetic route involves three enzymes and one spontaneous chemical reaction [42, 57]. First, glutamate kinase (ProB; EC 2.7.2.11) catalyzes the ATP-dependent phosphorylation of L-glutamate to form γ-glutamyl phosphate. This intermediate is then transformed by γ-glutamyl-phosphate reductase (ProA; EC 21.2.1.41) to glutamate-γ-semialdehyde, which spontaneously cyclizes to form Δ^1^-pyrroline-5-carboxylate. Finally, this compound is converted to L-proline by pyrroline-5-carboxylate reductase (ProC; EC 1.5.1.2) (Fig. 1b).

Given that the biosynthesis of a single L-proline molecule in bacteria consumes approximately 20 ATP equivalents [58], it is tightly regulated. In many microorganisms, the primary biochemical regulatory control point is the feedback inhibition of the ProB enzyme by the end-product of the pathway, L-proline [59–61]. Additionally, in some *Bacillus* species, the *proBA* and *proC* biosynthetic genes are transcriptionally regulated by a T-box riboswitch mechanism [62–64]. This L-proline-responsive riboswitch permits enhanced synthesis of full-length *proBA* and *proC* transcripts only under L-proline starvation conditions when growth has slowed [63], and when non-charged L-proline-specific tRNAs accumulate in the cell [62]. Both L-proline dependent feedback inhibition of ProB enzyme activity and T-box–mediated transcriptional regulation of anabolic *pro* genes prevents energetically costly [58] overproduction of L-proline. These combined biochemical and transcriptional regulatory set-points thus link the size of the cell’s cytoplasmic L-proline pool to the cell’s ongoing protein biosynthetic activities [60, 63].

L-proline is a widely used compatible solute in plants and bacteria that protects cells against high salinity and osmotic stress [42, 46, 65]. Under these conditions, substantial cytoplasmic L-proline pools are required to maintain cytoplasmic hydration, to preserve physiologically appropriate levels of turgor pressure, and to provide cytoprotection for essential cellular processes. The tight regulation of anabolic L-proline biosynthesis restricts L-proline accumulation under osmotic stress, thereby preventing the formation of the substantial L-proline pools necessary for cytoprotection [66, 67]. To overcome this constraint, several *members of the Bacillota* (e.g., *Bacillus subtilis*, *Bacillus licheniformis*, *Bacillus megaterium*) [64, 66, 68, 69] have evolved an alternative L-proline biosynthetic route that is specifically induced under osmotic stress, thereby enabling growth under these challenging environmental conditions (Fig. 1b). This osmotic stress-responsive L-proline biosynthetic route naturally bypasses both T-box-mediated transcriptional control and ProB feedback inhibition to attain large cytoplasmic L-proline pools whose size is sensitively linked to the degree of osmotic stress imposed onto the cell [64, 66, 69]. Of note: *B. megaterium*, a member of the *Bacillota*, has in the meantime been re-classified as *Priestia megaterium* [70] and this amended taxonomic description will be used in the following.

Due to the presence of multiple enzymes involved in L-proline biosynthesis in some *Bacillus* species, a revised nomenclature was introduced to distinguish between enzymes serving anabolic functions and those dedicated to osmotic stress adaptation [68]. In *B. subtilis*, the anabolic and osmotic stress-responsive biosynthetic pathways are interconnected via the shared use of the ProA enzyme [66, 68], whereas in *B. licheniformis* and *P. megaterium*, these pathways are genetically and biochemically separated (Fig. 1b) [64, 69]. Notably, *B. methanolicus* strain MGA3 [16] lacks the osmotic stress-responsive L-proline biosynthetic route, relying on the production of L-glutamate as its primary de novo-synthesized compatible solute [71].

Given that *B. methanolicus* MGA3 is a natural overproducer of L-glutamate under both osmotically balanced [23, 29] and high-salinity growth conditions [71], we hypothesized that this thermotolerant strain could potentially serve as a methanol-based chassis for the recombinant production of L-glutamate-derived L-proline. To this end, we constructed a synthetic operon using the *B. methanolicus* MGA3 anabolic L-proline biosynthetic *proBA* and *proI* genes as building-blocks, engineered to bypass the regulatory constraints imposed by the L-proline responsive T-box elements and ProB-mediated feedback inhibition. When established in *B. methanolicus* MGA3, this synthetic pathway enabled enhanced production and excretion of L-proline at high temperature in both shake-flask and fed-batch fermenter experiments.

## Materials and methods

### Bacterial strains

The *B. methanolicus* strain MGA3 (ATCC 53907) [15, 16] was provided by BASF SE (Ludwigshafen, Germany) for studies conducted at Marburg University (Germany) and by Acies Bio (Ljubljana, Slovenia). The *B. subtilis* strain JH642 (*trpC2 pheA1*; BGSC 1A96) [72] is derived from the domesticated laboratory strain *B. subtilis* 168 [73]. The *B. subtilis* strain JSB8 [Δ(*proHJ*::*tet*)] is a derivative of strain JH642 and is deficient in osmotic stress-adaptive L-proline biosynthesis but it is not a L-proline auxotroph [66]. The *B. licheniformis* strain DSM13 [64] was from our laboratory collection. The *Escherichia coli* strain MG1655 was used for all recombinant DNA experiments and its L-proline auxotrophic derivative strain MG1655-48 (*proC*::Tn*5*) (Pro^−^) [74] was used for the selection of Pro^+^ recombinant strains carrying plasmid-encoded functional L-proline biosynthetic gene clusters.

### Chemicals

Acetonitrile (HPLC grade) was purchased from VWR International GmbH (Heidelberg, Germany), and methanol (HPLC grade) was obtained from Carl Roth GmbH (Karlsruhe, Germany). The reagents *o*-phthaldialdehyde (OPA) and fluorenylmethyloxycarbonyl chloride (FMOC-Cl) used for amino acid derivatization for their subsequent analysis and quantification via HPLC analysis were purchased from Sigma-Aldrich (Taufkirchen, Germany). HPLC-grade standards for the quantification of amino acids (L-proline, L-glutamate, and L-citrulline) were also obtained from Sigma-Aldrich.

### Media and growth conditions

*B. subtilis* and *E. coli* strains were routinely maintained on LB agar plates [75], while *B. methanolicus* MGA3 was maintained on SOB agar plates [76]. For the growth of *B. subtilis*, a chemically defined medium, Spizizen’s Minimal Medium (SMM), supplemented with 0.5% (wt/vol) glucose and a trace element solution, was used [77]. To satisfy the auxotrophic requirements of the *B. subtilis* strain JH642 (*trpC2 pheA1*) [72], L-tryptophan (20 mg L⁻^1^) and L-phenylalanine (18 mg L⁻^1^) were added to SMM. *B. methanolicus* MGA3 was cultivated in MVcM [78], which consists of 23.3 mM K₂HPO₄, 10.8 mM NaH₂PO₄, 16 mM (NH₄)₂SO₄ (pH 7.8), a trace metal solution, D-biotin (0.1 mg L⁻^1^), and vitamin B₁₂ (0.01 mg L⁻^1^) [78]. Methanol (200 mM) was used as the sole carbon and energy source for cultivation of *B. methanolicus* MGA3-derived strains in MVcM using shake-flasks and an incubation temperature of 50° C. For *E. coli*, growth experiments were performed using minimal medium A (MMA) [75], supplemented with 0.5% (wt/vol) glucose, 1 mM MgSO₄, and 3 mM thiamine. *E. coli* strains carrying recombinant plasmids were cultivated in the presence of either chloramphenicol (30 µg mL⁻^1^) or ampicillin (100 µg mL⁻^1^), as required by the antibiotic resistance determinant encoded by the various types of plasmids used for functional studies. When recombinant plasmids were carried by *B. subtilis-* or by *B. methanolicus* MAG3-derived strains, kanamycin (10 µg mL⁻^1^) was added to the cultures. *E. coli* and *B. subtilis* cultures were grown at 37° C. Bacterial growth was monitored spectrophotometrically at a wavelength of 578 nm (OD₅₇₈).

A single colony of the *B. subtilis* strain JSB8 [66, 68] harboring different recombinant plasmids encoding L-proline biosynthetic genes was inoculated into 5 mL of LB medium and the culture was subsequently grown to mid-exponential phase. From this culture, 2 µL were transferred into 20 mL of SMM in a 100 mL Erlenmeyer flask; the culture was incubated overnight at 37° C in a rotating water bath set to 220 rpm to provide an adequate supply of oxygen for the *B. subtilis* cells. This pre-culture was used to inoculate, at an initial OD₅₇₈ of 0.1, the main *B. subtilis* cultures (20 mL in 100 mL Erlenmeyer flasks) for recombinant L-proline production; cells were grown at 37° C.

For L-proline production by *B. methanolicus* MGA3, a single colony was inoculated into 5 mL of SOB medium in 100 mL Erlenmeyer flasks with 200 mM methanol as the carbon and energy source and cultures were grown for 6 h to mid-exponential phase. Then, 15 µL of this pre-culture were transferred to 20 mL of MVcM containing 200 mM methanol and incubated overnight at 50° C in a rotating water bath (set to 220 rpm). These cultures were then used to inoculated, to an initial OD₅₇₈ of 0.1, all cultures for subsequent L-proline production experiments using methanol as the sole carbon and energy source; cultures were grown at 50° C. Details of the media composition used in bioreactors for L-proline production by a plasmid-carrying recombinant *B. methanolicus* MGA3 strain are specified in Table S1. All growth media were pre-warmed to the appropriate cultivation temperatures (37° C for *B. subtilis* and 50° C for *B. methanolicus* MGA3). The salinity of growth media was adjusted using a 5 M NaCl stock solution to the final concentrations specified in individual experiments.

### Construction of recombinant plasmids

An overview of the plasmids used in this study and their main characteristics are provided in Table 1.

**Table 1:** Plasmids used in this study

Plasmid	Functional characteristics	Reference/Source
pBV2mp^**1**^	*Bacillus/E. coli* shuttle vector; carries the P_*mdh*_ promoter; Kan^R^ and Amp^R^	[37]
pHSG575^**2**^	*E. coli* cloning vector allowing *lacZ* alpha-complementation; Cat^R^	[80]
pCF6	Derivative of pBV2mp lacking the P_*mdh*_ promoter	This study
pCF7	Derivative of pCF6 carrying the *B. licheniformis* DSM13 *proH-proJ-proAA* gene cluster under the transcriptional control of the native osmotically regulated P_*proHJAA*_ promoter. The *proJ* gene naturally carries an amino acid residue (R142) making the encoded enzyme resistant to feedback inhibition by L-proline	This study
pCF8	Derivative of pBV2mp *B. licheniformis* DSM13 *proH-proJ-proAA* gene cluster under the transcriptional control of both the native osmotically regulated P_*proHJAA*_ promoter and that of the *P*_*mdh*_ promoter. The *proJ* gene naturally carries an amino acid residue (R142) making the encoded enzyme resistant to feedback inhibition by L-proline	This study
pCF9	Derivative of pHSG575 carrying the synthetic *B. methanolicus* MGA3 *proB-proA-proI* gene cluster expressed under the transcriptional control of the P_*roBA*_ promoter; the T-box element is present	This study
pCF10	Derivative of pHSG575 carrying the synthetic *B. methanolicus* MGA3 *proB-proA-proI* gene cluster expressed under the transcriptional control of the P_*roBA*_ promoter; the T-box element was deleted	This study
pCF11	Derivative of pCF10 carrying the synthetic *B. methanolicus* MGA3 proB*-proA-proI gene cluster expressed under the transcriptional control of the P_*roBA*_ promoter; the T-box element was deleted. The proB* allele (E142/R) was generated through site-directed mutagenesis and confers feedback resistance of ProB by L-proline	This study
pCF12	This *Bacillus/E. coli* shuttle plasmid carries the synthetic *proB-proA-proI* gene cluster from *B. methanolicus MGA3* under the transcriptional control of the P_*mdh*_ promoter; the T-box element was deleted. The *proB* gene encodes a feedback-sensitive ProB enzyme	This study
pCF13	This *Bacillus/E. coli* shuttle plasmid carries the synthetic *proB-proA-proI* gene cluster from *B. methanolicus MGA3* under the *t*ranscriptional control of the P_*proBA*_ promoter; the T-box element was deleted. The *proB* gene encodes a ProB enzyme feedback-sensitive to L-proline	This study
pCF21	This plasmid carries the synthetic *proB-proA-proI* gene cluster from *B. methanolicus MGA3* under the transcriptional control of the P_*mdh*_ promoter; the T-box element was deleted. The proB* gene encodes a ProB enzyme feedback-resistant to L-proline. This construct is carried by the pBV2mp *Bacillus/E. coli* shuttle vector	This study
pCF22	This plasmid carries the synthetic *proB-proA-proI* gene cluster from *B. methanolicus MGA3* under the transcriptional control of the native P_*proBA*_ promoter; the T-box element was deleted. The proB* gene encodes a ProB enzyme feedback-resistant to L-proline. This construct is carried by the pBV2mp *Bacillus/E. coli* shuttle vector	This study

^1^Plasmid pBV2mp as a *Bacillus/E.coli* shuttle vector and carries genes encoding resistance against the antibiotics kanamycin (Kan^R^) and ampicillin (Amp^R^)

^2^Plasmid pHSG575 is a low-copy number *E. coli* cloning vector carries a gene encoding resistance against the antibiotic chloramphenicol (Cat^R^)

To construct a promoter-less *Bacillus/E. coli* shuttle vector, the constitutive *B. methanolicus* MGA3 methanol dehydrogenase (*mdh*) promoter present on plasmid pBV2mp [37] was removed using the restriction enzymes XbaI and SpeI, which generate compatible overhangs. This digestion produced two DNA fragments (with length of 1,046 bp and 6,736 bp, respectively). The 6,736 bp DNA fragment was re-ligated using T4 DNA ligase (Thermo Fisher Scientific Inc., Waltham, MA, USA), yielding the promoter-less *Bacillus/E. coli* shuttle vector pCF6.

For the construction of a plasmid carrying the *proH-proJ-proAA* osmotic stress-adaptive L-proline biosynthetic operon from *B. licheniformis* DSM13 [64], the corresponding gene cluster was amplified by PCR using chromosomal DNA from *B. licheniformis* DSM13 as a template and the primers 11_proHJAA_CF7_fw and 12_proHJAA_CF7_rv (Table S2). The resulting PCR product and DNA from the KpnI-linearized plasmid pCF6 were assembled using the Gibson Assembly method [79], resulting in plasmid pCF7.

Plasmid pCF8 was generated by amplifying the *proH-proJ-proAA* operon from *B. licheniformis* DSM13, including its native osmotically inducible promoter (P*proHJAA*) [64] using primers 10_proHJAAGi_fw and 12_proHJAA_CF7_rv (Table S2). Chromosomal DNA from *B. licheniformis* DSM13 served as the PCR template. The amplified fragment was ligated with KpnI-linearized DNA from plasmid pBV2mp using Gibson Assembly [79] to produce plasmid pCF8.

The *B. methanolicus* MGA3 anabolic L-proline biosynthetic genes *proBA* and *proI* are located at different positions on the chromosome. Both the *proBA* operon and the *proI* gene is predicted to be transcriptionally controlled by T-box riboswitches (Fig. S1a, b and S2).

To construct a synthetic anabolic L-proline biosynthetic gene cluster from *B. methanolicus* MGA3 [16], the *proBA* operon was amplified using primers P89_proBA_pHSG_fw and P90_proBA_pHSG_rv; the *proI* gene (without the T-box element) was amplified using primers P91_proI_pHSG_fw and P92_proI_pHSG_rv (Table S2). The resulting two PCR fragments were inserted into the low-copy-number plasmid pHSG575 (Cm^r^) [80] via Gibson Assembly [79], yielding plasmid pCF9, which carries the complete synthetic *proBA-proI* operon for anabolic L-proline biosynthesis. In this plasmid, the DNA-segment of the *proI* gene encoding the T-box regulatory element was removed and fused to the 3’-end of the *proBA* operon using a short synthetic DNA-linker sequence. An overview on the synthetic *proB-proA-proI* operon from *B. methanolicus* MGA3 is provided in Fig. S3.

Deletion of the T-box regulatory element naturally positioned upstream of the *proBA* genes (Fig. S1a) in *B. methanolicus* MAG3 was performed using the Q5® Site-Directed Mutagenesis Kit (New England BioLabs GmbH, Frankfurt, Germany). DNA of plasmid pCF9 served as the template, and primers P123_del_TF and P124_delT2F (Table S2) were used for mutagenesis, resulting in plasmid pCF10. The *proB* gene carried by this plasmid encodes a ProB enzyme that is sensitive to feedback inhibition by L-proline.

To reduce (or abolish) the presumed allosteric inhibition of the *B. methanolicus* MGA3 anabolic ProB enzyme by L-proline, a mutation causing a single amino acid substitution (E142/R) [59–61] was introduced into the *proB* gene using the Q5® Site-Directed Mutagenesis Kit. Primers P97_Q5_proB_E142R_fw and P98_Q5_proB_E142R_rv (Table S2) were used with DNA of plasmid pCF10 as the template, resulting in plasmid pCF11. The mutated gene/protein is denoted as proB^*^/ProB* throughout this manuscript.

Plasmid pCF12 is based on the *Bacillus/E. coli* shuttle vector pBV2mp and carries the synthetic L-proline *proB-proA-proI* biosynthetic gene cluster from *B. methanolicus* MGA3. It is expressed under the control of the *mdh* promoter but the DNA segment encoding the T-box element (Fig. S3) was removed. The *proB* gene encoded by this plasmid carries a natural *proB* allele (E142) that confers sensitivity to feedback regulation of ProB enzyme activity by L-proline.

Plasmid pCF13 carries the same L-proline biosynthetic gene cluster with the removed T-box element (Fig. S3), and it also carries a natural *proB* allele (E142) that causes sensitivity to feedback regulation of ProB enzyme activity by L-proline. This gene cluster is expressed from the native *proBA* promoter of *B. methanolicus* MGA3.

To construct an expression system for L-proline biosynthesis under control of the *B. methanolicus* MGA3 *mdh* promoter and a *proB* allele (R142) conferring feedback resistance of the ProB enzyme by L-proline, the synthetic proB^*^-proA-proI operon was amplified from plasmid pCF11 using primers P126_ProS and P127_ProS2 (Table S2). DNA of the pBV2mp vector (carrying the *mdh* promoter) was linearized with KpnI, and the two DNA fragments were assembled via Gibson assembly [79] to yield plasmid pCF21. In this plasmid, the DNA segment encoding the T-box element positioned upstream of the *proB-proA* genes (Fig. S1 and Fig. S3) was removed through mutagenesis using primers P124_del-T2F and P123_del-TF (Table S2).

For the construction of a L-proline biosynthetic operon lacking the T-box regulatory region (Fig. S3) (P*proBAΔTBox*) and carrying a *proB* allele (R142) conferring feedback resistance of ProB to L-proline, we constructed a plasmid in which this operon was expressed from the native *B. methanolicus* MGA3 *proBA* promoter. Accordingly, DNA from plasmid pCF11 was amplified using primers proIWT2_pCF6_fw and proIWT2_pCF6_rv (Table S2) and cloned into the linearized plasmid pCF6 via Gibson assembly [79]. The resulting plasmid was designated pCF22. The synthetic *proB-proA-proI* operon from *B. methanolicus* MGA3 is expressed from the native P_*proBA*_ promoter.

In all cloning procedures, recombinant strains capable to synthesize L-proline were selected by transforming the assembled plasmids into cells of the proline-auxotrophic *E. coli* strain MG1655-48 (*proC::Tn5*) [74]. Accordingly, transformants were plated on MMA minimal agar plates [75] lacking L-proline to exploit the L-proline auxotrophic phenotype (Pro^−^) of this strain for the selection of Pro^+^ derivatives expressing recombinant plasmid-based functional L-proline biosynthetic gene clusters.

### Recombinant L-proline production in a fed-batch fermenter system

Fermentative production of L-proline by *B. methanolicus* MGA3 under high-temperature (50° C) fed-batch conditions was carried out in a 5 L bioreactor (Satorius Biostat B) with methanol as the carbon and energy source essentially as previously described [23]. The *B. methanolicus* MGA3 (pCF21) L-proline production cell factory was grown in two replicas in bioreactors with a starting volume of 2.5 L under fed-batch methanol cultivation conditions. The bioreactor seed culture was prepared in two stages. First, cultures were inoculated directly from glycerol stocks [20% glycerol in water; stored at -20° C] into 250 mL Erlenmeyer flasks containing 25 mL of standard SOBman media (Table S1) and incubated for 8–10 h at 50° C and 180 rpm. The second stage was inoculated with a 10% (V/V) inoculum size into 2L Erlenmeyer flasks filled with 200 mL of MVcMy media (Table S1). The second stage cultures were also incubated at 50° C and were grown at 180 rpm until an OD_600_ of 2–2.5 was reached (4–5 h). Aliquots of this pre-culture were subsequently used to inoculate the bioreactor equipped with a 5-L glass vessel and filled with 2.5 L of ABBM-PM-BR media (Table S1) using an inoculum size of 10% (V/V), pre-warmed to 50° C.

The dissolved oxygen concentration in the bioreactor was maintained at about 30% via automatic adjustment of the stirrer speed. Methanol was continuously fed to maintain a constant concentration of about 9 g L⁻^1^ in the growth medium. Methanol feed was added by a peristaltic pump controlled by an Arduino microcontroller. The controller was connected to a silicone tubing methanol sensor submerged into the fermentation vessel [81]. The methanol feed also contained 50 mL L^−1^ of CKNFD trace elements (Table S1).

As *B. methanolicus* MGA3 cells are known to excrete considerable amounts of the negatively charged amino acid L-glutamate [23, 29], the pH of the culture in the bioreactor was held at 6.5 through automated addition of 20% ammonium hydroxide, which also served as the nitrogen source for the cells. During the fermentation run, the OD_600_ and pH values were monitored continuously. Samples were withdrawn periodically to determine cell dry weight (CDW) (g L⁻^1^) and culture osmolarity (mOsmol kg⁻^1^). Intracellular and extracellular amounts of L-glutamate and L-proline were quantified by HPLC analysis. Fermentations were carried out for 40 h until the cultures reached stationary phase, resulting in a maximum CDW of 46.4 ± 3 g L⁻^1^. Two independent fermentation runs were performed, and key cultivation parameters are summarized in Table S3.

### HPLC analysis of amino acids

For the quantification of amino acids, *B. methanolicus* MGA3 and *B. subtilis* JSB8, each carrying various recombinant plasmids encoding L-proline biosynthetic genes, or the corresponding empty cloning vector, were cultivated in MVcM or SMM, respectively, until mid-exponential phase (OD₅₇₈ ≈ 1.5). From each culture, 10 mL was harvested by centrifugation at 2,500 × *g* for 10 min at room temperature. The supernatant was collected and immediately stored at –20° C. Low-molecular-weight intracellular compounds were extracted from the cell pellet using a modified Bligh and Dyer method as previously described [82].

For quantitative amino acid analysis, both cell extracts and supernatants were derivatized with *o*-phthaldialdehyde (OPA) and fluorenylmethyloxycarbonyl chloride (FMOC-Cl) using an automated procedure based on the method of Krömer et al. [83]. Briefly, a 0.5 µL sample was incubated with 0.5 µL of FMOC (1.25 mg mL⁻^1^ in acetonitrile) for 1 min at room temperature. Next, 0.5 µL of OPA reagent [10 mg mL⁻^1^ in a 1:75:75 (v/v/v) mixture of 2-mercaptoethanol [2-MCE], methanol, and 0.4 M borate buffer, pH 10.2] was added and incubated for 1 min at room temperature. The reaction mixture was then diluted by the addition of 36 µL H₂O and injected into an HPLC system (1260 Infinity, Agilent Technologies, Waldbronn, Germany) equipped with a 150 × 4.6 mm Gemini C18 column (5 µm, 110 Å; Phenomenex, Aschaffenburg, Germany). Amino acid separation was achieved using a gradient elution protocol with solvent A (40 mM phosphate buffer, pH 7.8) and solvent B (acetonitrile-methanol–water, 45:45:10, v/v/v). The gradient profile was as follows. 0 min: 0% B—40.5 min: 40.5% B—43 min: 61% B—44 min: 82% B—46.5 min: 100% B—47 min: 0% B. The flow rate was set to 1 mL min⁻^1^, and the column temperature was maintained at 40° C. Detection and quantification of labeled amino acids were performed using a fluorescence detector (Agilent Technologies). L-glutamate and L-citrulline were detected at an excitation wavelength of 304 nm and an emission wavelength set to 450 nm, while L-proline was detected at 266 nm (excitation) and 305 nm (emission) wavelengths. Amino acid concentrations were determined by comparing the HPLC data with appropriate commercially available standards of L-glutamate, L-proline, and L-citrulline. Data acquisition and analysis were performed using the OpenLab software suite (Agilent Technologies).

### Determination of the osmolarity of growth media and culture supernatants

During fed-batch methanol-based fermentation of *B. methanolicus* MGA3 (pCF21) in a 5-L bioreactor, the osmolarity of both the starting growth medium and culture supernatants during the fermentation process was measured at selected time intervals using a freezing point osmometer (Osmomate 3000; Gonotec, Berlin, Germany).

### Computer analysis and modelling of protein structures

The DNA sequences of the core genes involved in L-proline metabolism [63, 66, 68, 84] were retrieved from the genome sequence of the *B. subtilis* strain JH642 [72] and used to identify homologous genes in the *B. methanolicus* MGA3 genome sequence [16]. The presence of a T-box transcriptional regulatory mechanism [62, 63] in *B. methanolicus* MGA3 was investigated by aligning the 250 bp DNA-sequence positioned upstream either of the *proBA* operon, or that of the *proI* gene, (Fig. S1 a, b)) with the corresponding regions present in *B. subtilis* JH642 that contain the experimentally verified T-box elements of the *proBA* operon and of the *proI* gene of *B. subtilis* [63], using the MAFFT online alignment server (https://mafft.cbrc.jp/alignment/server/) [85]. Specific sequence features of the T-box motif and related elements [62, 63] were additionally examined manually. The possible secondary structures of the *proBA* and *proI* mRNA leader transcripts from *B. methanolicus* MGA3 were predicted using the Mfold program (http://mfold.rna.albany.edu/) [86] and were further refined through manual curation based on established T-box structural models [62, 63] and are depicted in Fig. S2.

An in silico structural model of the *B. methanolicus* MGA3 ProB glutamate-kinase was generated using the SWISS-MODEL server (https://swissmodel.expasy.org/) [87], which automatically selected the crystal structure of the *E. coli* ProB protein [Protein Data Base (PDB) accession codes: 2j5v and 2j5t] [88] as the most suitable template. Graphical representations of both the *E. coli* ProB crystal structure and the in silico generated model of the *B. methanolicus* MGA3 ProB protein were generated using the PyMOL molecular visualization system (https://pymol.org/2/).

## Results

### in silico analysis of the *B. methanolicus* MGA3 genome sequence for L-proline biosynthetic and degradative genes

We analyzed the genome sequence of *B. methanolicus* strain MGA3 [16] for genes involved in L-proline biosynthesis and catabolism, using corresponding *B. subtilis* genes as references [66, 68, 84]. Homologs of the anabolic *proBA* and *proI* biosynthetic genes were identified (Fig. 1a), with amino acid sequence identities of 66% (ProB), 65% (ProA), and 51% (ProI) compared to the corresponding proteins from *B. subtilis*. No genes for osmotic stress-adaptive L-proline biosynthesis such as found in *B. subtilis*, *B. licheniformis* and *B. megaterium*/*P. megaterium* [64, 66, 69, 70] were detected in the *B. methanolicus* MAG3 genome sequence (Fig. 1a, b), consistent with its exclusive use of newly synthesized L-glutamate as a compatible solute under osmotic stress conditions [71].

**Fig. 2:**
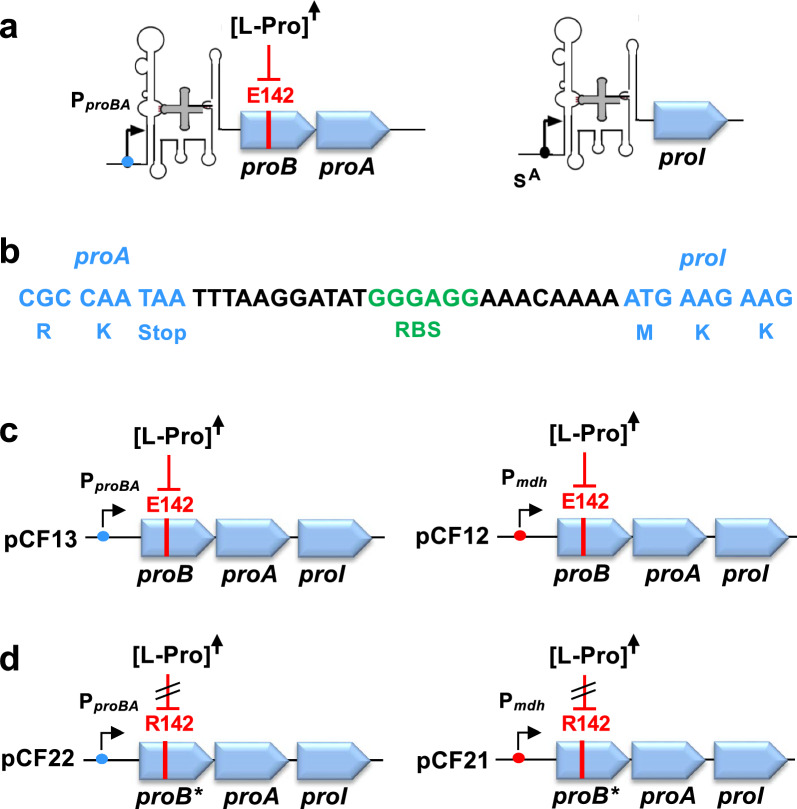
Genetic organization of anabolic L-proline biosynthetic genes in *B. methanolicus* MGA3 and design of a synthetic L-proline biosynthesis operon. (**a**) In *B. methanolicus* MGA3, *proBA* and *proI* are located at separate loci on the genome and are controlled by predicted L-proline–responsive T-box riboswitches. The ProB enzyme is predicted to be feedback-inhibited by L-proline via the conserved E142 residue near the active site [60, 61]; increased intracellular L-proline (upward-pointing arrow) thus inhibits ProB enzyme activity. (**b**) A synthetic *proB–proA–proI* operon was constructed by fusing the *proI* coding region to the 3′ end of the *proBA operon*, including a synthetic 25-bp intergenic region with a putative ribosome-binding-site (RBS). (**c, d**) The operon was expressed from either the native *proBA* promoter (pCF13, pCF22) or the *mdh* promoter (pCF12, pCF21), with native T-box elements removed. The various *proB* genes encode either feedback-sensitive ProB enzymes (E142) or predicted feedback-resistant ProB*-type enzymes (R142) [59]

Transcription of the *B. subtilis proBA* and *proI* anabolic genes is regulated by T-box riboswitches [63] thereby coordinating L-proline synthesis with the overall translational activity of the cell [62]. We detected similar type riboswitches in the 5′-UTRs of the *proBA* and *proI* from *B. methanolicus* MGA3 (Fig. 2a; Fig. S1 and S2) and found that the predicted secondary structures of the corresponding T-box riboswitches, including the presence of CCU-L-proline specifier codons [62, 63], resemble those present in *B. subtilis* (Fig. S2) [63]. As L-proline-responsive *proBA*- and *proI*-type T-box riboswitches function as negative-acting transcriptional control elements, anabolic L-proline production in *B. methanolicus* MGA3 is only increased beyond basal levels when the steady-state cytoplasmic L-proline pool is inadequate to support the ongoing cellular protein biosynthesis. [62, 63].

**Fig. 3:**
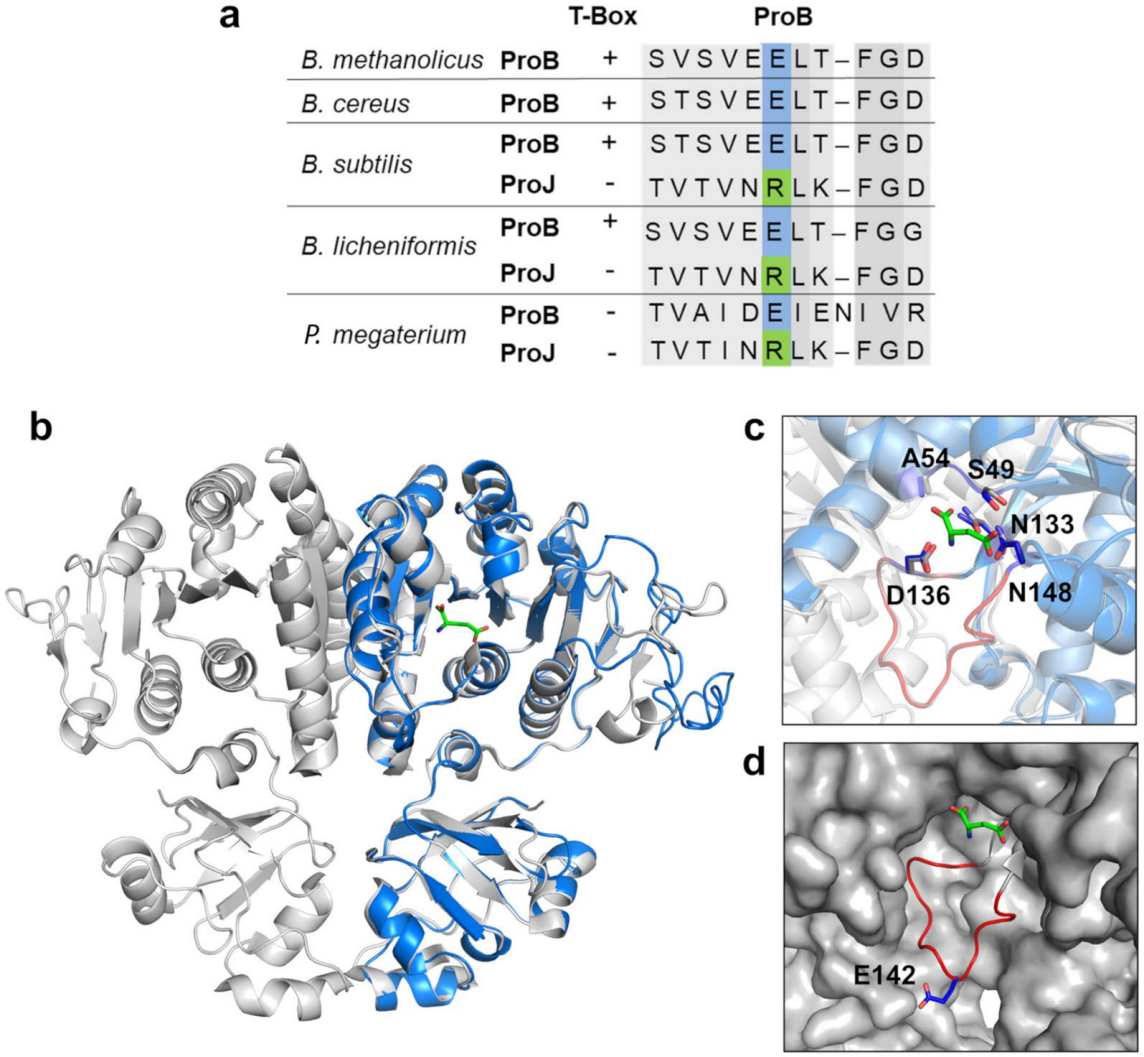
Structural predictions and sequence analysis of glutamate 5-kinase (ProB) from *B. methanolicus* MGA3. (**a**) Alignment of amino acid sequences of predicted feedback-sensitive ProB-type and feedback-resistant ProJ-type enzymes from *Bacillus cereus*, *Bacillus subtilis*, *Bacillus licheniformis* and *Priestia megaterium*, highlighting residue 142: E142 in ProB (blue) and R142 in ProJ (green). (**b**) Superimposition of the modeled ProB monomer from *B. methanolicus* MGA3 (blue) with the dimeric ProB protein from *E. coli* as captured in its crystal structure (PDB files: 2j5v and 2j5t; grey) bound to L-glutamate (sticks). (**c**) Structural elements involved in L-proline feedback inhibition and L-glutamate binding [60, 61, 88]. L-glutamate is shown as green sticks and a 16 amino acid residues long regulatory loop of ProB is highlighted in red. (**d**) Residue E142, critical for L-proline feedback control, is located on the flexible loop positioned adjacent to the substrate-binding site [61]; the ProB protein surface shown in grey

Beyond transcriptional control via the T-box mechanisms, enzyme activity of the anabolic ProB protein is frequently subject to feedback inhibition by L-proline [42, 59, 89], involving a substrate-effector site overlap and a regulatory mobile loop carrying key amino acids for feedback control (Fig. 3) [60, 61, 88]. In the *B. methanolicus* MGA3 ProB protein, the corresponding critical amino acid residue is a L-glutamate residue (E142) positioned on the mobile loop (Fig. 3a). Substitution of this negatively charged L-glutamate residue with a positively charged L-arginine residue (E → R) typically confers resistance of the ProB enzyme to feedback inhibition by L-proline [59–61, 88]. This is naturally observed in ProJ-type enzymes involved in osmotic stress-adaptive synthesis of L-proline by *B. subtilis*, *B. licheniformis*, and *P. megaterium*, and the corresponding feedback-responsive ProB anabolic enzymes (Fig. 3a) [59, 64, 66, 69].

*B. subtilis* can exploit imported L-proline as a nutrient via the activities of the PutP L-proline importer and the associated cytoplasmic catabolic enzymes PutB and PutC [84]. The corresponding genes, along with that of the L-proline-responsive transcriptional activator protein PutR/PrcR [90, 91], are genetically organized into a *putB-putC-putP-putR* gene cluster in *B. subtilis* [84]. Transcription of the *putB-putC-putP* operon is inducible by externally provided L-proline in a PutR/PrcR-dependent fashion, where *putR/*prcR is expressed from its own promoter [91]. Corresponding genes are not present in the *B. methanolicus* MAG3 genome sequence [16]. There are however three separately positioned genes encoding proteins possessing 59% amino acid sequence identity (BMMGA3_14425) to the *B. subtilis* PutB protein, a protein with 72% amino acid sequence identity (BMMGA3_01740) to PutC, and a protein with 26% amino acid sequence identity (BMMGA3_13490) to the PutP transporter. However, these proteins are seemingly not involved in L-proline utilization as a nutrient, as our growth data show that *B. methanolicus* MGA3 cannot use this amino acid either as sole carbon, energy, or as nitrogen source (Fig. S4 a, b). Of note, it also cannot use the proteogenic amino acids L-Glu, L-Asp, L-Arg, L-Gln and the non-proteogenic amino acids L-Orn, and L -Cit as sole carbon and energy source (Fig. S4 a, b). This is also true for the use of these amino acids when supplied as sole nitrogen sources, except for L-Gln which can be utilized by *B. methanolicus* MGA3 as a nitrogen source (Fig. S4 a, b).

### Expression of osmotic stress-adaptive L-proline genes from *B. licheniformis* in *B. methanolicus* MGA3 boost L-citrulline production, not L-proline biosynthesis

Our in silico analysis of the anabolic L-proline biosynthetic pathway in *B. methanolicus* MGA3 indicates that L-proline production is regulated at both the transcriptional and post-transcriptional levels. While these regulatory features are physiologically beneficial for the fine-tuning for the energy-demanding anabolic L-proline production [58], they limit the utility of these native biosynthetic genes for engineering a thermotolerant L-proline over-production strain. Therefore, we focused initially on heterologous L-proline biosynthetic gene clusters from osmotic stress-adaptive pathways present in Gram-positive bacteria such as *B. subtilis*, *B. licheniformis* and *B. megaterium*/*P. megaterium* [59, 64, 66, 69, 70], as these L-proline biosynthetic pathways naturally lack T-box regulation and encode ProB variants predicted to be resistant to feedback inhibition (Fig. 1b and Fig. 3a).

**Fig. 4:**
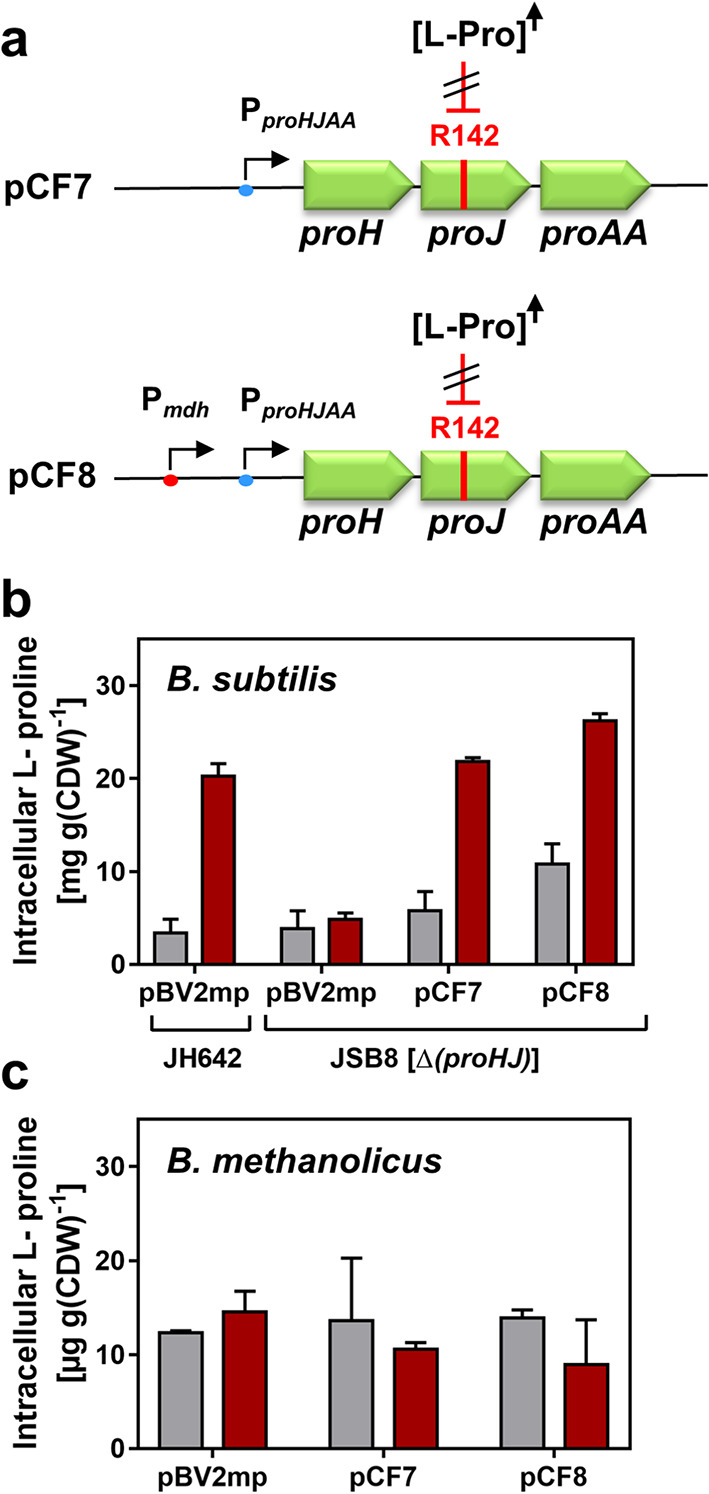
Introduction of the osmotic stress–adaptive *proH–proJ–proAA* operon from *B. licheniformis* DSM13 into *B. subtilis* JSB8 and *B. methanolicus* MGA3. **a** Genetic organization of the osmotic stress–adaptive *proH–proJ–proAA* operon from *B. licheniformis* DSM13 [64] carried on plasmids pCF7 and pCF8. Both plasmids contain the feedback-resistant *proJ* allele (E142R). Expression of the operon is driven by the native osmotic stress responsive promoter in plasmid pCF7 [64], and additionally by the constitutive *mdh* promoter from *B. methanolicus* MGA3 in plasmid pCF8. **b** Intracellular L-proline levels in *B. subtilis* JH642 (wild type) and *B. subtilis* JSB8 [Δ(*proHJ::tet*)], defective in the osmotic stress-responsive L-proline biosynthetic route [66]. Cultures were grown in SMM (grey) or SMM + 0.5 M NaCl (red) at 37° C with glucose as the caron and energy source to OD₅₇₈ ≈ 1.5; L-proline was quantified by HPLC analytics. **c** L-proline levels in *B. methanolicus* MGA3 carrying the plasmid-encoded *B. licheniformis proH–proJ*–*proAA* cluster. Cultures were grown in MVcM in the absence (grey bars) or the presence of 0.5 M NaCl (red bars) at 50° C on methanol as the carbon and energy source to OD₅₇₈ ≈ 1.5; L-proline was quantified by HPLC analytics. In (**b**) and (**c**), data represent the mean ± SD of three independent biological replicates, each measured in duplicate. Cultures of *B. methanolicus* MGA3 cells carrying the empty cloning vector pBV2mp was used as reference for the native L-proline content of the cells

To this end, we used the osmotic stress-adaptive *proH-proJ-proAA* L-proline biosynthetic gene cluster from *B. licheniformis* DSM13 (Fig. 1b) [64] for heterologous expression in *B. methanolicus* MGA3. We constructed two plasmids, pCF7 and pCF8**,** each carrying the *B. licheniformis proH-proJ-proAA* operon. In both constructs, the L-proline biosynthetic operon is expressed from its native osmotically regulated *proHJAA* promoter [64], while pCF8 additionally includes the constitutive *mdh* promoter from *B. methanolicus* MGA3 [16] to drive expression of the heterologous gene cluster. The *proJ* gene carried by these two plasmids are predicted to naturally encode ProJ-type feedback resistant glutamate kinases (Fig. 4a).

We separately introduced these plasmids, and the empty vector pBV2mp [37] by DNA transformation, into the *B. subtilis* strain JSB8, a derivative of the wild-type strain JH642 in which the osmotic stress-adaptive L-proline biosynthetic route has been genetically disrupted [66, 68]. As expected from the osmotic regulation of the *B. licheniformis* L-proline biosynthetic operon through its transcriptional control via the *proHJAA* promoter and lack of feedback control (Fig. 1B; Fig. 4a) [64], the presence of either plasmid pCF7 or pCF8 restored the ability of the osmotically sensitive *B. subtilis* JSB8 mutant strain to produce L-proline under osmotic stress conditions (in SMM medium supplemented with 0.5 M NaCl) (Fig. 4b). The recombinant strains produced L-proline at levels comparable to those found in the *B. subtilis* wild-type strain JH642 harboring the empty vector pBV2mp. This strain depends on its endogenous osmotic stress–adaptive *proJ–proA–proH* biosynthetic pathway (Fig. 1b) to synthesize L-proline at physiologically appropriate levels that mitigate the detrimental effects of osmotic stress on cellular physiology and growth (Fig. 4b) [66]. The presence of the additional and constitutively active *mdh* promoter from *B. methanolicus* MGA3 on plasmid pCF8 (Fig. 4a) increased L-proline production in the heterologous host *B. subtilis* moderately under both non-osmotic and osmotic stress conferring growth conditions when compared with the *B. subtilis* strain that expressed the *proH-proJ-proAA* operon only from the authentic *proHJAA* promoter present on plasmid pCF7 (Fig. 4b). Collectively, these results demonstrate that the osmotic stress-adaptive *proH-proJ-proAA* operon from *B. licheniformis* DSM13 [64] can be functionally expressed from plasmids pCF7 and pCF8 in *B. subtilis* thereby producing osmotic stress-adaptive L-proline pools in the heterologous host strain.

**Fig. 5:**
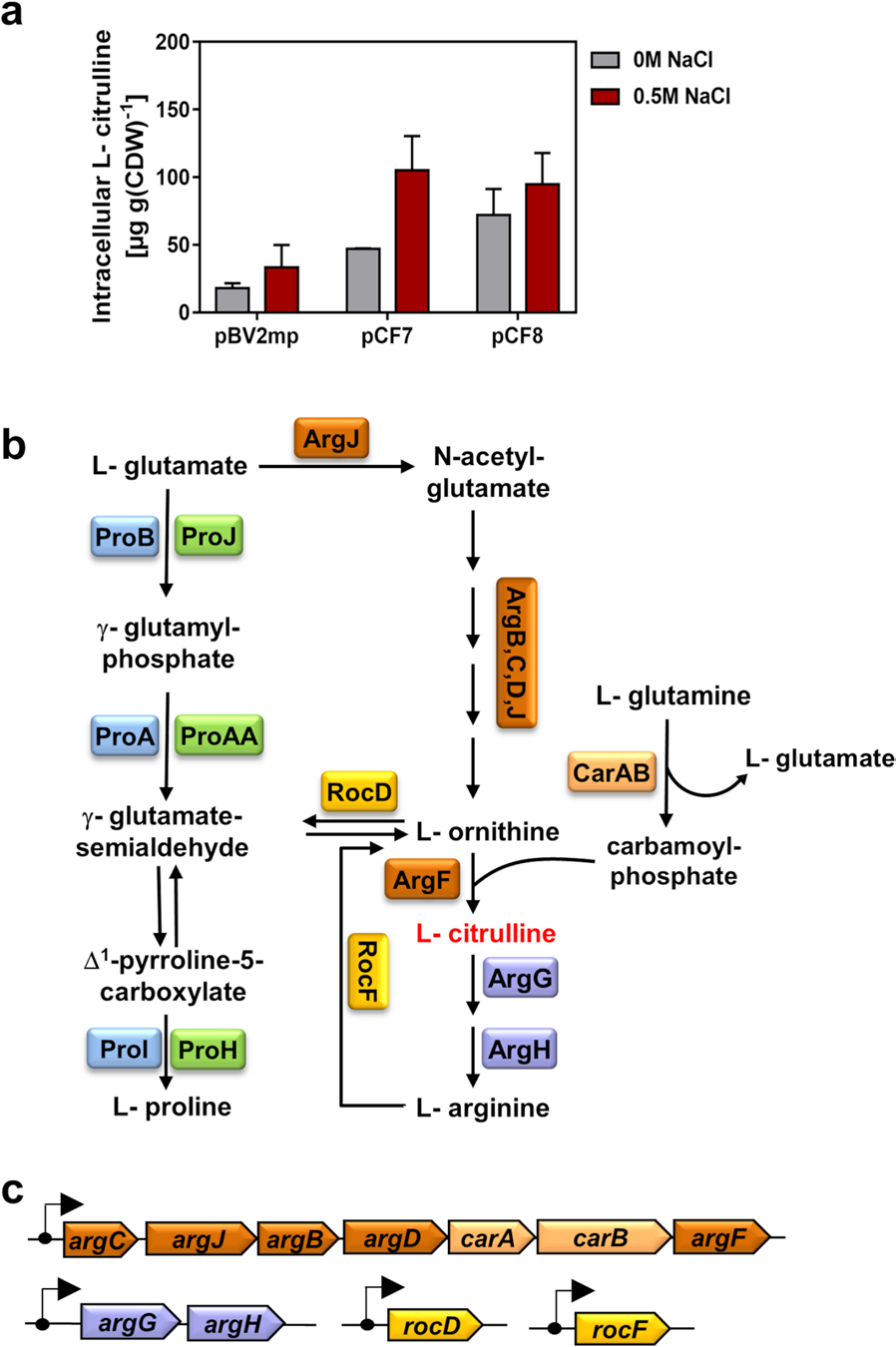
Redirection of osmotic stress adaptive L-proline production toward L-citrulline synthesis in *B. methanolicus* MGA3. (**a**) *B. methanolicus* MGA3 cells expressing the osmotic stress–adaptive *proH–proJ–proAA* genes from *B. licheniformis* DSM13 [64] were grown at 50° C in MVcM with methanol as the carbon and energy source, without or with 0.5 M NaCl. Plasmid pCF7 expresses the L-proline biosynthetic gene cluster from the native osmotic stress-responsive promoter, while pCF8 also uses the constitutive *mdh* promoter. Both plasmids carry the feedback-resistant *proJ* (E142R) allele. Cells were harvested at an OD₅₇₈ ≈ 1.5, and intracellular L-citrulline was quantified by HPLC analytics. Grey bars: MVcM; red bars: MVcM + 0.5 M NaCl. Data are means of two biological replicates, each measured in duplicate. (**b**) Predicted core metabolic network for L-proline and L-arginine biosynthesis in *Bacillus* spp. [92]. (**c**) Genetic organization of L-arginine biosynthetic operons in the *B. methanolicus* MGA3 genome [16]; arrows indicate predicted promoters

However, unexpectedly, introduction of plasmids pCF7 or pCF8 into *B. methanolicus* MGA3 did not result in increased intracellular L-proline levels when the strains were cultivated at 50° C with methanol as the carbon and energy source, either in absence or presence of 0.5 M NaCl in the growth medium (Fig. 4c). This result prompted us to investigate the underlying cause. HPLC analysis of cell extracts from *B. methanolicus* MGA3 carrying plasmids pCF7 or pCF8 revealed elevated levels of intracellular L-citrulline (Fig. 5a), an intermediate in the L-arginine biosynthesis pathway in various Bacilli (Fig. 5 b, c) [92–94]. L-citrulline production by recombinant *B. methanolicus* MGA3 cells was osmotically inducible (Fig. 5a), thereby reflecting the osmotic control of the *B. licheniformis proHJAA* promoter [64] that is present on both plasmids pCF7 and pCF8 (Fig. 4a). The additional presence of the constitutive *mdh* promoter present on plasmid pCF8 led to an increase in the intracellular L-citrulline pools in the absence of osmotic stress, while its influence on L-citrulline production under osmotic stress-conferring conditions was negligible (Fig. 5a). Quantitatively, the L-citrulline concentration increased from about 18 µg g^−1^ dry cell weight (CDW) in the control strain (*B. methanolicus* MGA3 harboring the empty cloning vector pBV2mp) grown in the absence of additional NaCl to about 105 µg g^−1^ CDW in the strains carrying either plasmid pCF7 or pCF8 when the cells were grown in the presence of 0.5 M NaCl (Fig. 5a). This corresponds to an approximately fivefold increase in the steady-state pool-size of intracellular L-citrulline in the recombinant *B. methanolicus* MGA3 cell factory.

One plausible explanation for the observed lack of L-proline accumulation in the recombinant thermotolerant *B. methanolicus* MGA3 strain is the potential temperature sensitivity of the Δ^1^-pyrroline-5-carboxylate reductase ProH derived from the mesophile *B. licheniformis* DSM13 (Fig. 1b) [64]. If this assumption is correct, this should lead to the accumulation of the L-proline biosynthetic intermediate γ-glutamate-semialdehyde (Fig. 5b). The chemically highly reactive γ-glutamate-semialdehyde interconnects the L-proline and L-arginine biosynthesis routes in various Bacilli [92–94]. Accordingly, enzyme activities of RocD from the L-arginine biosynthetic rout (Fig. 5b) can channel γ-glutamate-semialdehyde toward L-ornithine formation, which can subsequently be converted to L-citrulline by the ArgF enzyme (Fig. 5b) [92–94]. The corresponding genes encoding these enzymes are present in the *B. methanolicus* MGA3 genome sequence (Fig. 5c) [16].

### Design of a synthetic L-proline biosynthetic operon comprising the anabolic *proBA* and *proI *genes from *B. methanolicus* MGA3

Our attempt to exploit the *proH-proJ-proAA* operon from the mesophilic *B. licheniformis* for L-proline production in the thermotolerant *B. methanolicus* MGA3 strain was ultimately unsuccessful. Consequently, we revised our strategy for L-proline overproduction and shifted our focus toward the anabolic L-proline biosynthetic genes native to *B. methanolicus* MGA3 (Fig. 1a), as the corresponding enzymes must be inherently adapted to the organism’s thermophilic lifestyle [15–17].

To render the native *proBA* and *proI* genes suitable for engineered L-proline overproduction, it was necessary to overcome their natural genetic and biochemical regulatory constraints that limit L-proline production. Specifically, this required eliminating transcriptional control by T-box riboswitch elements (Fig. 2a; Fig. S1 and S2) and circumventing the presumed feedback inhibition of the activity of the *B. methanolicus* anabolic ProB enzyme by L-proline (Fig. 3). As the *proBA* and *proI* genes are located at separate positions on the *B. methanolicus* MGA3 genome [16], we employed recombinant DNA techniques to construct a synthetic *proB-proA-proI* operon, fusing the three genes required for L-proline biosynthesis into a single transcriptional unit (Fig. 2b) (Fig. S3a). In this construct, the transcriptional T-box regulatory elements located upstream of the native *proBA* and *proI* coding regions (Fig. S1a,b and Fig. S3b, c) were removed to eliminate their L-proline-responsive negative effects on the full-length transcription of the synthetic *proH-proJ-proAA* operon when the cellular L-proline pools are high [63].

To evaluate the effect of transcriptional regulation on L-proline production, we placed this synthetic construct under two different promoters: (i) the native *proBA* promoter from *B. methanolicus* MGA3, and (ii) the constitutive *mdh* promoter from this species (Fig. 2c). Furthermore, to eliminate potential feedback inhibition of the anabolic ProB enzyme by L-proline, we performed site-directed mutagenesis to substitute the codon encoding Glu142 (E142) with a codon for Arg (R), generating a presumed feedback-resistant variant that we termed ProB* (Fig. 2d and Fig. 3a). This specific E142/R amino acid substitution has previously been shown to either strongly reduce or abolish feedback inhibition of ProB-type enzymes [59–61].

**Fig. 6:**
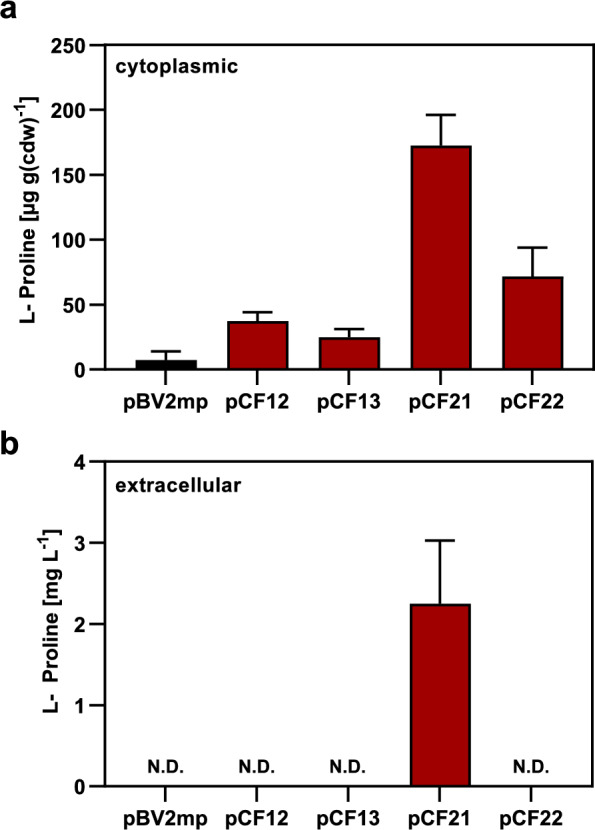
Functional expression of a synthetic L-proline biosynthetic gene cluster in *B. methanolicus* MGA3. *B. methanolicus* MGA3 strains expressing synthetic *proB–proA–proI* (feedback-sensitive ProB, E142; pCF13, pCF22) or proB*–*proA–proI* (feedback-resistant ProB*, R142; pCF12, pCF21) operons under the native *proBA* promoter (pCF13, pCF22) or the constitutive *mdh* promoter (pCF12, pCF21) are shown. Cultures were grown in MVcM at 50° C on methanol as carbon and energy source to an OD₅₇₈ ≈ 1.5, and intracellular (**a**) and extracellular (**b**) L-proline levels were measured by HPLC analytics. Data represent means ± SD of two biological replicates, each measured in duplicate. N.D.: not detected. *B. methanolicus* MGA3 carrying the empty cloning vector pBV2mp served as empty vector control

Collectively, we created through molecular engineering, four plasmids that express different versions of the synthetic *proB-proA-proI* L-proline biosynthetic operon (Fig. 2c, d), each of which lack the DNA-segment encoding the T-box transcriptional regulatory element. Two plasmids express the native *proB-proA-proI* operon (encoding a feedback-sensitive ProB enzyme) (plasmids pCF12 and pCF13), and two plasmids express a modified proB*-proA-proI operon (encoding a feedback-resistant ProB* enzyme) (plasmids pCF21 and pCF22). Each of these synthetic operons is controlled by either the *proBA* or the *mdh* promoter. These plasmids enabled us to systematically investigate how (i) the type of promoter (*proBA* vs. *mdh*), and (ii) the ProB enzyme variant (sensitive vs. resistant to feedback inhibition by L-proline) affect L-proline production in recombinant *B. methanolicus* MGA3 high-temperature cell factories (Fig. 6a).

### L-proline production by *B. methanolicus* MGA3 cultures grown in shake flasks

To this end, plasmids pCF12**,** pCF13**,** pCF21**,** and pCF22 were separately transformed into *B. methanolicus* MGA3 and the intra- and extracellular L-proline pools were evaluated via HPLC analysis. The corresponding cultures were grown in synthetic minimal MVcM medium with methanol as sole carbon and energy source at 50° C in 100-ml Erlenmeyer flasks containing 20 ml of culture volume**.** After approximately 10 h of growth**,** the cultures reached an optical density at 578 nm (OD₅₇₈) of about 1.5. Cells were then harvested by centrifugation, and both cellular extracts and culture supernatants were analyzed for their L-proline content**.**

As a baseline, cells harboring the empty vector (pBV2mp) contained an intracellular L-proline pool of approximately 8 µg g^−1^ CDW (Fig. 6a), reflecting the native L-proline anabolic biosynthetic activity of *B. methanolicus* MGA3. Expression of the synthetic *proB-proA-proI* operon under the control of the native *proBA* promoter (plasmid pCF13; Fig. 2c) led to a 4.6-fold increase in intracellular L-proline levels, reaching 37 µg g^−1^ CDW. When the gene cluster encoding the feedback-resistant ProB* variant was expressed under the control of the same promoter (plasmid pCF22; Fig. 2d), L-proline accumulation increased further to 72 µg g^−1^ CDW, representing a ninefold enhancement compared to the *B. methanolicus* MGA3 (pBV2mp) control strain (Fig. 6a). Since both plasmids use the *proBA* promoter, these results underscore the significant contribution of the feedback-resistant ProB* enzyme to enhanced L-proline production in the recombinant cell factory.

A similar trend was observed when the synthetic L-proline biosynthetic operon was expressed under the control of the *mdh* promoter using the recombinant plasmids pCF12 (carrying the *proB* gene) (Fig. 2c) and pCF21 (carrying the proB* gene) (Fig. 2d). The *mdh*-driven synthetic L-proline biosynthetic operon present on pCF21 led to higher L-proline pools compared with those established in cells carrying plasmid pCF22 in which this operon is expressed under the control of the *proBA* promoter. The best performing cell factory [*B. methanolicus* MGA3 (pCF21) (*mdh* promoter and presence of the proB* allele)] contained an intracellular L-proline pool of 172 µg g^−1^ CDW, corresponding to a 21-fold increase over the basal level observed in *B. methanolicus* MGA3 (pBV2mp) (Fig. 6a) and about a 2.4-fold increase compared with cells expressing the synthetic operon expressing the proB* allele under the control of the native *proBA* promoter [*B. methanolicus* MGA3 (pCF22)].

Collectively, the presence of the feedback-resistant ProB* enzyme (encoded by plasmid pCF21) made a substantial contribution to the steady-state L-proline pool. It was also apparent that the *mdh* promoter was more efficient in driving the transcription of the synthetic proB*-proA-proI operon in comparison with cells expressing the same gene cluster under the control of the *proBA* promoter (Fig. 6a). Collectively, these results demonstrate that integrating a feedback-resistant ProB* enzyme with a strong constitutive promoter (*mdh*) yields an optimal high-temperature–tolerant *B. methanolicus* MGA3 cell factory for the overproduction of L-proline.

### The best-performing recombinant *B. methanolicus* MGA3 cell factory excretes L-proline

*B. methanolicus* MGA3 naturally secretes large amounts of L-glutamate into the growth medium [23, 29]. This secretion surprisingly persists even under osmotic stress-conferring growth conditions, when the intracellular accumulation of the compatible solute L-glutamate is required to maintain physiological adequate levels of cellular hydration and turgor pressure [71]. Given this background information, we investigated whether the engineered *B. methanolicus* MGA3 strain might also secrete L-proline. Indeed, our best-performing recombinant strain, *B. methanolicus* MGA3 (pCF21) carrying the synthetic proB*-proA-proI gene cluster under the transcriptional control of the *mdh* promoter, excreted L-proline into the culture medium (Fig. 6b). After 10 h of growth in shake flasks, the culture supernatant of this strain contained 2.25 mg L^−1^ L-proline. In contrast, neither the wild-type strain carrying the empty vector (pBV2mp), nor the recombinant strain expressing the proB*-proA-proI operon under the control of the *proBA* promoter showed detectable amounts of L-proline in the culture medium (Fig. 6b). Collectively, these data suggest that recombinant *B. methanolicus* MGA3 cells must accumulate cytoplasmic L-proline pools above a critical threshold relative to the native intracellular pool prior to initiating secretion of this amino acid into the growth medium.

### High salinity does not enhance recombinant production of L-proline

Markedly higher intracellular and extracellular levels of L-glutamate are produced when *B. methanolicus* MGA3 is challenged by high salinity/osmolarity [71]. Because L-glutamate is the direct biosynthetic precursor for L-proline [42, 57], we hypothesized that salt-induced L-glutamate accumulation might further enhance L-proline production in the engineered *B. methanolicus* MGA3 strains. Contrary to expectation, L-proline accumulation in the best-performing cell factory [*B. methanolicus* MGA3 (pCF21)] (Fig. 6a) was repressed to a significant degree under elevated salinity conditions**,** when the growth medium was supplemented with either 0.3 M or 0.4 M NaCl (Fig. S5). The molecular and physiological mechanism underlying this salt-dependent repression of *mdh*-driven L-proline biosynthesis remains unclear and warrants further investigation. The negative effects of high salinity on L-proline production may result from inhibitory impacts of altered intracellular ionic strength and macromolecular crowding [95] on *mdh* promoter activity as well as/or on the activity of enzymes involved in L-proline biosynthesis [42].

### L-proline production and excretion by recombinant *B. methanolicus* MGA3 under fed-batch conditions

To further evaluate the performance of the *B. methanolicus* MGA3 L-proline production cell factory, we cultivated our best-performing recombinant strain carrying plasmid pCF21 (Fig. 6a) in a 5-L bioreactor with an initial medium volume of 2.5-L. Cells were grown at 50° C in a modified MVcM minimal medium supplemented with D-biotin and yeast extract. Methanol was continuously fed to maintain a constant concentration of about 9 g L⁻^1^ in the growth medium. The pH in the bioreactor was held at 6.5 through automated addition of 20% ammonium hydroxide to the medium. The OD₅₇₈ and pH values were monitored continuously during the fermentation run, and the rotor speed was adjusted to provide an adequate oxygen supply for the cells. Samples were withdrawn from the bioreactor periodically to determine cell dry weight (g L⁻^1^) and culture osmolarity (mOsmol kg⁻^1^). Intracellular and extracellular concentrations of L-glutamate and L-proline were quantified by HPLC analysis. Fermentations were carried out for 40 h resulting in a maximum CDW of 46.4 ± 3 g L⁻^1^. Two independent fermentation runs were performed, and key cultivation parameters are given in Table S3.

**Fig. 7:**
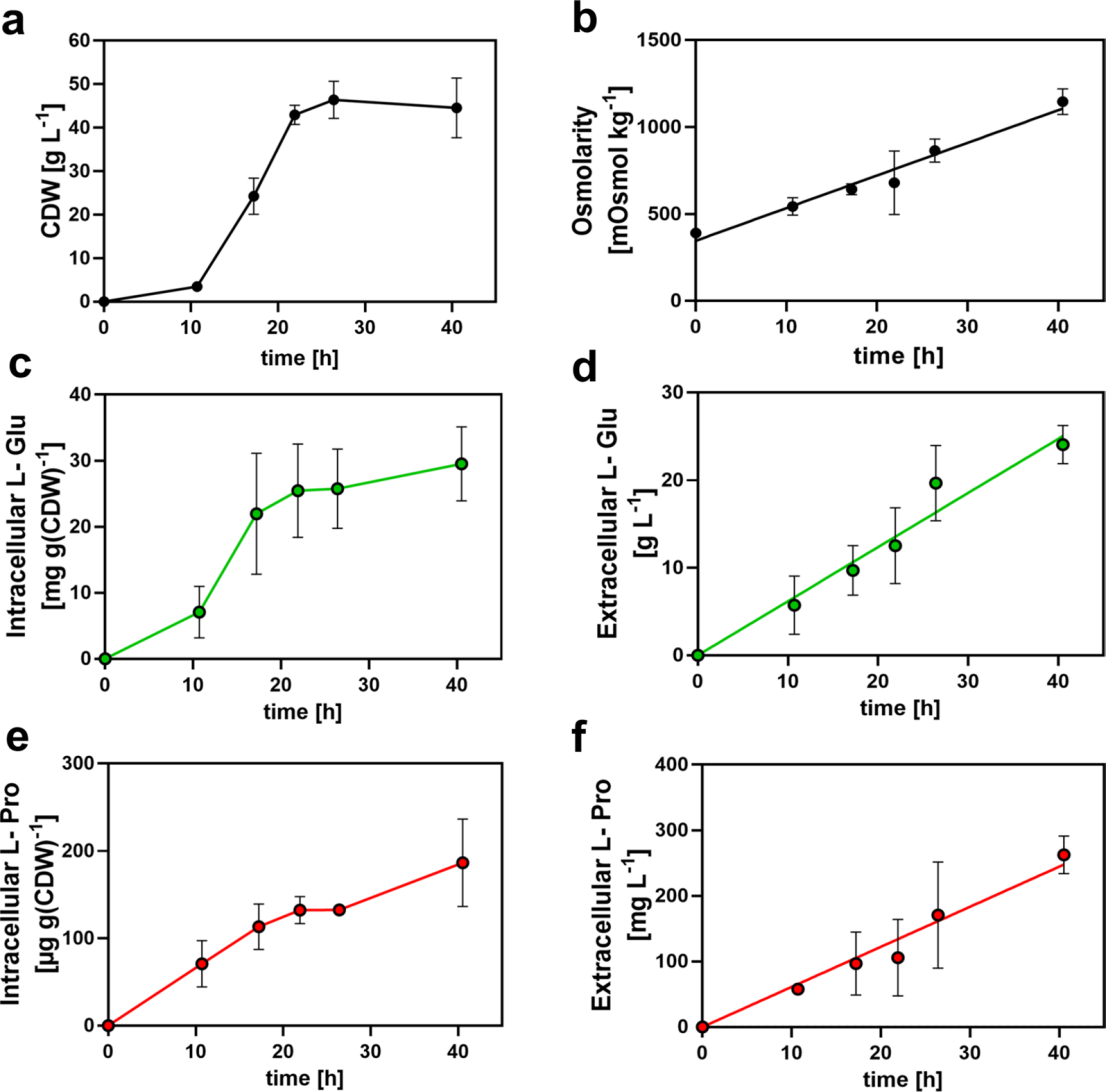
Production and excretion of L-proline by the *B. methanolicus* MGA3 (pCF21) cell factory under fed-batch conditions with methanol as the carbon and energy source. *B. methanolicus* MGA3 carrying plasmid pCF21 (P_*mdh*_-proB*-*proA–proI*) was grown at 50° C in a 5 L fermenter with methanol maintained during the fermentation process at 9 g L⁻^1^. Samples were collected at indicated time points to measure cellular dry weight (CDW) (**a**), culture medium osmolarity (**b**), and intracellular (**c, e)** and extracellular (**d, f**) L-glutamate (L-Glu) and L-proline (L-Pro) pools by HPLC analytics

As summarized in Fig. 7, both cultures of *B. methanolicus* MGA3 (pCF21) in the bioreactor showed a steady biomass accumulation (Fig. 7a), accompanied by an essential linear increase in the osmolarity of the growth medium (from 392 mOsmol kg⁻^1^ to 1136 mOsmol kg⁻^1^) (Fig. 7b). The intracellular and extracellular concentrations of L-glutamate (Fig. 7c, d) and L-proline (Fig. 7e, f) increased throughout the growth phase. Maximal intracellular levels of L-glutamate reached 29 ± 4 mg g⁻^1^ CDW (Fig. 7c) and 166 ± 55 µ g⁻^1^ CDW for L-proline (Fig. 7e). The extracellular concentrations of L-glutamate and L-proline reached about 22.6 g L⁻^1^ (Fig. 7d) and 262 ± 20 mg L⁻^1^ of (Fig. 7f), respectively. Hence, compared with the shake-flask experiment where the L-proline pool reached about 2 mg L^−1^ (Fig. 6b), the extracellular L-proline pool increased in the bioreactor experiment, even if one takes the longer fermentation time (40 h versus 10 h) and different growth parameters of the shake-flasks and reactor experiment into account.

## Discussion

This study presents an integrative strategy to enhance recombinant L-proline production in *B. methanolicus* strain MGA3 from the environmentally friendly feedstock methanol. This natural methylotroph is an emerging thermotolerant cell factory for energy-efficient, low-carbon manufacturing of value-added fine-chemicals and proteins [17–19, 27, 96]. For heterologous overproduction of L-proline in *B. methanolicus* MGA3, we employed either genes encoding an osmotic stress–responsive L-proline biosynthetic pathway from the mesophile *B. licheniformis* DSM13, as this type of pathway is expected to support high-level L-proline production [64, 66, 67], or genes encoding the native anabolic L-proline biosynthetic pathway of *B. methanolicus* MGA3, since the corresponding enzymes are likely adapted to the thermotolerant lifestyle of this methylotrophic bacterium [16].

While implementation of the heterologous L-proline biosynthetic pathway was ultimately unsuccessful, leveraging the native anabolic pathway in *B. methanolicus* MGA3 proved effective for L-proline production. Increased L-proline production was achieved by building on the robust L-glutamate overproduction capacity of *B. methanolicus* MGA3 [23, 29] and by relieving biochemical and transcriptional constrains within its native *proBA*- and *proI*-encoded anabolic L-proline biosynthetic (Fig. 1a; Fig. S1-S3). Collectively, our results demonstrate the feasibility of establishing a thermotolerant, methanol-based *B. methanolicus* MGA3 cell factory for L-proline production and secretion. Although L-proline is currently produced at substantially lower industrial volumes than other amino acids [1–3], its nutritional, cytoprotective, and pharmaceutical relevance [42–44, 97], as well as its role as a precursor for commercially valuable hydroxyprolines [55, 56], has fostered growing interest in its biotechnological production [49–54].

L-proline is an important compatible solute that is widely utilized by microorganisms to counteract the adverse effects of high osmolarity on turgor, cellular physiology and growth [46, 67, 97, 98]. Osmotic stress-responsive L-proline biosynthetic routs present in multiple members of the *Bacillota* (e.g., *B. subtilis*, *B. licheniformis*, *P. megaterium*) [64, 66, 69, 70] are characterized by relief from biochemical feedback inhibition of the ProB enzyme by L-proline and L-proline-responsive T-box-mediated transcriptional repression otherwise limiting the energetically costly overproduction of L-proline [42, 58]. Contrary to expectations, heterologous expression of such an osmotic stress-responsive L-proline biosynthetic gene cluster obtained from the mesophile *B. licheniformis* DSM13 in the thermotolerant *B. methanolicus* MGA3 strain did not boost L-proline production but serendipitously triggered L-citrulline accumulation (Fig. 5a).

This effect is not completely understood at the biochemical and physiological level but is likely caused by the heat sensitivity of ProH from *B. licheniformis* DSM13, a Δ^1^-pyrroline-5-carboxylate reductase (Fig. 1b), together with metabolic crosstalk at the shared intermediate γ-glutamate-semialdehyde. This metabolite connects the L-proline and L-arginine biosynthetic pathways in various Gram-positive bacteria (Fig. 5b) [92–94, 99]. In the engineered *B. methanolicus* MGA3 strain, the metabolic shunt formed around γ-glutamate-semialdehyde is expected to promote excess L-ornithine production when the last step in L-proline production is blocked through the activity of the RocD ornithine aminotransferase. In a subsequent step, the ArgF enzyme (ornithine transcarbamylase; OCT) converts L-ornithine into L-citrulline [92, 99]. The further metabolic flux from L-citrulline to L-arginine, mediated by the enzyme of the AhrC-repressed *argG-argH* operon (Fig. 5b, c), is likely constrained by a highly integrated regulatory network involving both positive and negative transcriptional regulators, including RocR, AhrC, and SigL, as well as by the intracellular pool sizes of key effector molecules such as L-ornithine, L-citrulline, and L-arginine [93, 94, 99–101]. Although the precise mechanisms underlying L-citrulline accumulation in the engineered *B. methanolicus* MGA3 strain remain to be fully elucidated, our results highlight the role of this methylotroph as a versatile and promising platform for amino acid production [23, 36]. This is particularly significant in the context of our study because L-citrulline is a commercially valuable non-proteinogenic amino acid [102, 103],

Given the limited success of the osmotic inducible heterologous L-proline biosynthetic system, we shifted our focus to the native anabolic L-proline biosynthetic pathway of *B. methanolicus* MGA3, as its enzymes are expected to be inherently thermotolerant [15–17]. To overcome L-proline-mediated ProB feedback inhibition and T-box-limited transcription of the *proBA* and *proJ* genes (Fig. 2a), we engineered a synthetic *proBA–proI* operon not subjected to these regulatory constraints (Fig. S3). Expression of the engineered operon in *B. methanolicus* MGA3 under the control of the constitutive *mdh* promoter increased intra- and extracellular L-proline levels (Fig. 6a). By comparing L-proline production of this strain with a corresponding strain carrying a feedback-sensitive *proB* allele under the same promoter (Fig. 6a), our data highlight the combined importance of feedback deregulation (compare pCF12 with pCF21; Fig. 2c) and transcriptional strength (compare pCF21 with pCF22; Fig. 2c) for recombinant L-proline overproduction by *B. methanolicus* MGA3.

Although the titers currently attained in this study in laboratory-scale fermenters remain modest (Fig. 7e, f), our *proof-of-concept* study demonstrates the feasibility of engineering *B. methanolicus* MGA3 for methanol-based L-proline production and secretion at elevated temperatures (50 °C). The achieved extracellular L-proline titers [262 ± 20 mg L^−1^ over a fermentation time of 40 h] (Fig. 7f) are substantially lower than those reported for the biotechnological workhorse *Corynebacterium glutamicum*, in which extensive metabolic engineering has enabled L-proline production titers reaching up to 142 g L⁻^1^ [49, 53]. Nonetheless, *B. methanolicus* MGA3 remains an interesting platform for biotechnology [17–19, 96] as it offers the advantage over other microbial cell factories that it can naturally utilize methanol, a sustainable and inexpensive C_1_-feedstock [5–8], instead of sugars [49–51, 53, 54] for L-proline bioproduction.

In our experiments, methanol-fed batch fermentation of *B. methanolicus* MGA3 at 50° C led to the secretion up to 22.5 g L⁻^1^ of L-glutamate within 40 h (Fig. 7d), while recombinant strains produced substantially lower levels of extracellular L-proline (262 ± 20 mg L⁻^1^) in the same timeframe (Fig. 7f). The observed proportionality between intra- and extracellular L-glutamate and L-proline pools (Fig. 7c, d, e, f) indicates that L-proline secretion by *B. methanolicus* MGA3 is not per se limited by export capacity by the cell. Recent work by Brito et al*.* [29] identified the sole MscS-type mechanosensitive channel operating in *B. methanolicus* MGA3 [16] as the primary mediator of L-glutamate efflux (Fig. 1a). Similarly, the MscS-type mechanosensitive channel MscCG mediates release of L-glutamate during steady-state growth in *C. glutamicum* [104–107]. Although the MscS-type channels involved in L-glutamate release in *C. glutamicum* and *B. methanolicus* MGA3 belong to different MscS subfamilies [108], as judged by distinct transmembrane architectures [29, 105], they share common functional characteristics [29]. MscS-type channels in bacteria typically function as excess turgor-relief valves activated by sudden osmotic downshifts via a *force-from-lipid* mechanism and they usually lack substrate specificity [109]. It should be noted in this context, that a dedicated L-proline exporter has recently also been discovered in *C. glutamicum* [53]. However, the molecular mechanisms underlying the export of L-proline from the recombinant cell factory that we describe here for *B. methanolicus* MGA3 remain to be elucidated (Fig. 1a).

**Fig. 8:**
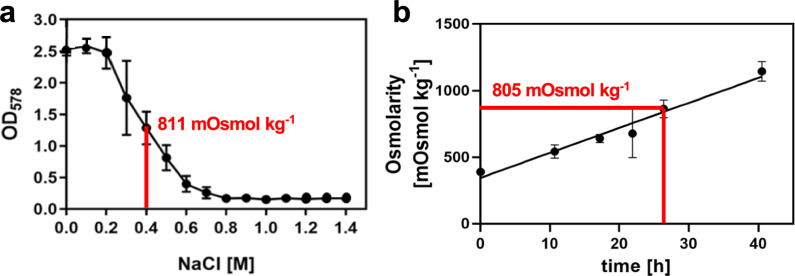
Comparison of the growth profile of salt-stressed *B. methanolicus* MGA3 in shake-flask cultures with the increase in medium osmolarity during fed-batch fermentation. **a** Data from Frank et al. [71] (see Fig. 1B from this publication) were replotted. The red bar indicates the medium osmolarity causing 50% growth reduction in salt-stressed shake-flask cultures. **b** Osmolarity during methanol-based fed-batch fermentation of *B. methanolicus* MGA3 (pCF21) (this study, Fig. 7b) replotted for direct comparison with the data of Frank et al*.* [67]. The red bar indicates the approximate 50% growth inhibition osmolarity. Data from Frank et al*.* [67] are reproduced under the CC BY license, allowing unrestricted use with proper attribution

Excessive L-glutamate secretion during methanol-based fermentation of *B. methanolicus* MGA3 (Fig. 7d) lowers medium pH and increases osmolarity of the growth medium (Fig. 7b), both of which will inhibit growth. While pH can readily be controlled in a fermenter system, adjustment of osmolarity during fed-batch cultivation of *B. methanolicus* MGA3 is more problematic [71]. In our fed-batch experiments in a laboratory-scale bioreactor, medium osmolarity rose from 392 to 1146 mOsm kg⁻^1^ during the fermentation run over 40 h (Fig. 7b), a condition known to restrict growth of *B. methanolicus* MGA3 in shake-flasks cultures (Fig. 8) [71]. Unlike *B. subtilis*, which synthesizes L-proline as a reasonably efficient osmotic stress-protectant [66, 110], *B. methanolicus* MGA3 relies solely on the synthesis of the less-effective compatible solute L-glutamate [97, 110]. Consequently, *B. methanolicus* MGA3 cannot withstand true high-salinity environments [71], despite its ability to grow in sea-water [28]. It also cannot use externally provided compatible solutes such as glycine betaine or rely on the hydrolysis of L-proline-containing peptides present in components of rich media for osmotic stress relief as observed in *B. subtilis* [67, 111]. Thus, increasing osmolarity likely contributed to growth cessation in late fermentation stages of *B. methanolic* MGA3 cultures (Fig. 8), even as amino acid secretion continued (Fig. 7). This effect results in a desirable decoupling of biomass production from L-proline biosynthesis and secretion.

In summary, our work establishes a thermotolerant, methanol-based platform for L-proline production through rational engineering of the *B. methanolicus* MGA3’s native anabolic biosynthetic pathway. The absence of L-proline catabolism in *B. methanolicus* MGA3 (Fig. S4a, b), in contrast to the situation found in *B. subtilis* [84], and the organism’s inherent thermotolerance [15, 16] make it an attractive chassis for further optimization for L-proline overproduction and secretion. Collectively, our findings expand the metabolic engineering potential of *B. methanolicus* MGA3 as an emerging cell factory for low-carbon manufacturing of value-added products [17–19, 27] and highlight its promise for sustainable, C_1_-based biomanufacturing of the amino acids L-proline and L-citrulline.

## Supplementary Information

Supplementary Material 1.

Supplementary Material 2.

Supplementary Material 3.

Supplementary Material 4.

## Data Availability

The original data presented in this study are included in the article/ Supplementary information; further inquiries can be directed to the corresponding author (E.B.).

## Abbreviations

LB: Luria Bertani medium
MMA: Minimal Medium A
SMM: Spizizen’s Minimal Medium
PCR: Polymerase Chain Reaction
rpm: Revolutions per minute
Amp^r^: Ampicillin resistance
Kan^r^: Kanamycin resistance
Cml^r^: Chloramphenicol resistance
CDW: Cell dry weight
ProA: γ-Glutamyl-phosphate reductase
ProB: Glutamate kinase sensitive to feedback inhibition by L-proline
ProJ: Glutamate kinase resistant to feedback inhibition by L-proline
ProI and ProH: Iso-enzymes of pyrroline-5-carboxylate reductase
