# Supplementary material for: Harnessing the thermotolerant methylotroph Bacillus methanolicus for methanol-based synthetic L-proline production
Source: Microb Cell Fact. 2026 May 23;25:129. doi: 10.1186/s12934-026-03032-8 (PMC13198045; doi:10.1186/s12934-026-03032-8)
Supplement: Supplementary file 1 — Supplementary Material 1. [file 12934_2026_3032_MOESM1_ESM.pdf]

Supplementary figures for:

Harnessing the thermotolerant methylotroph *Bacillus methanolicus* for methanol-based synthetic L-proline production

Christine Frank[1], David Virant[2], Gregor Kosec[2], Tamara Hoffmann[1,3], Oskar Zelder[4], Max F. Felle[4], and Erhard Bremer[1,3,*]

[1]Faculty of Biology, Microbiology, Marburg University, D-35043 Marburg, Germany

[2]Acies Bio d.o.o, Tehnoloski park 21, SI-1000 Ljubljana, Slovenia

[3]Center for Synthetic Microbiology (SYNMIKRO), Marburg University, D-35043 Marburg, Germany

[4]BASF SE, RGR/D – A030, D-67056 Ludwigshafen am Rhein, Germany

*Corresponding author (bremer@staff.uni-marburg.de)

E-mails:

Christine Frank: christine.schwarzkopf07@gmx.de

David Virant: david.virant@aciesbio.com

Gregor Kosec: gregor.kosec@aciesbio.com

Tamara Hoffmann: hoffmant@staff.uni-marburg.de

Oskar Zelder: oskar.zelder@basf.com

Max F. Felle: max-fabian.felle@basf.com

Erhard Bremer: bremer@staff.uni-marburg.de

____

***Correspondence to Dr. Erhard Bremer:** Marburg University, Center for Synthetic Microbiology (SYNMIKRO), Karl-von-Frisch Strasse 14, D-35043 Marburg, Germany. Phone: (+49)-6421-2821529. E-Mail: bremer@staff.uni-marburg.de

Figure S1: Regulatory region of the *proBA* and *proI* anabolic L-proline biosynthetic genes from *Bacillus methanolicsus MGA3.*

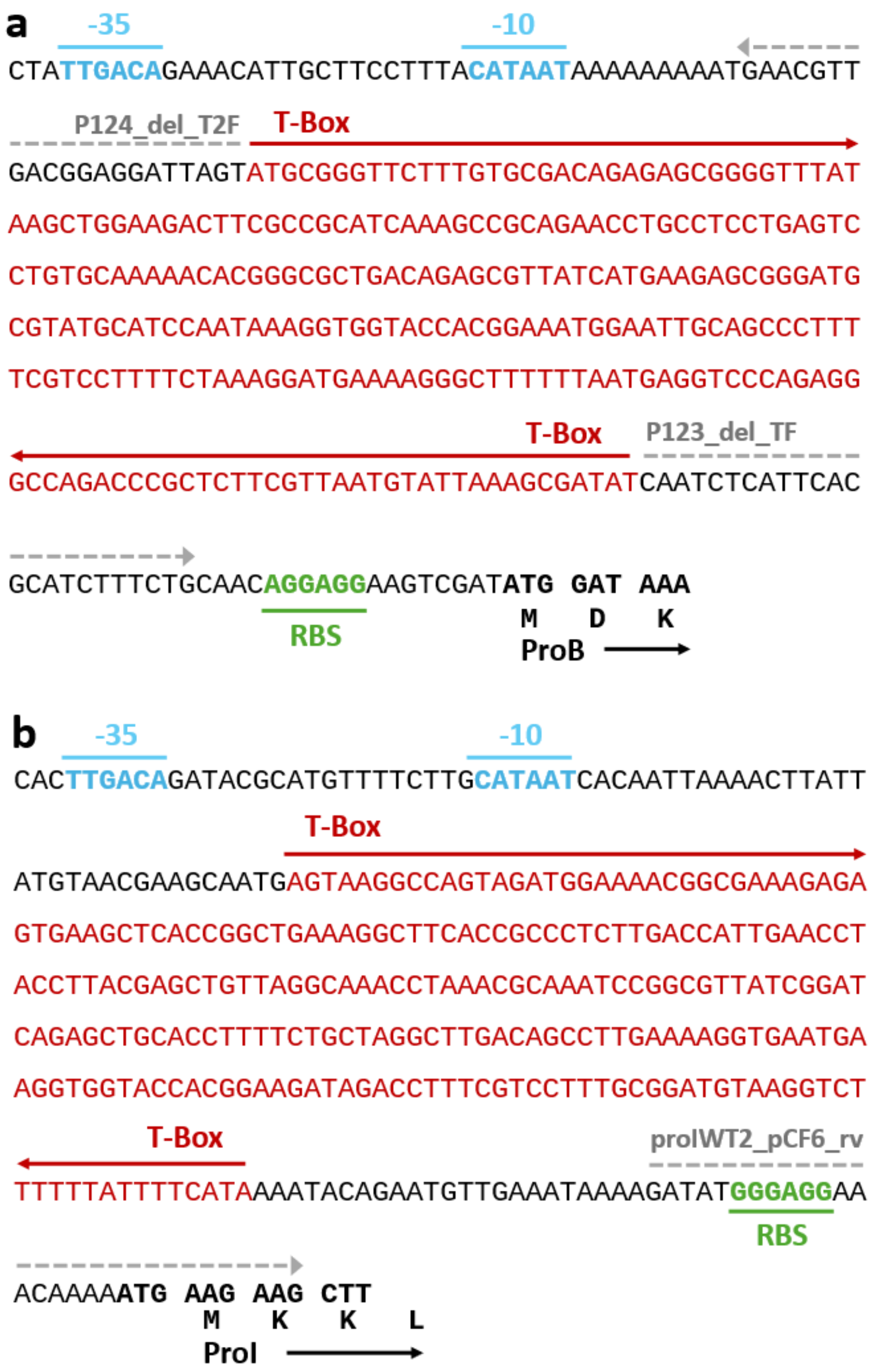
a
-35
-10
CTATTGACAGAAACATTGCTTCCTTTACATAATAAAAAAAAATGAACGTT
P124_del_T2F
T-Box
GACGGAGGATTAGTATGCGGGTTCTTTGTGCGACAGAGAGCGGGGTTTAT
AAGCTGGAAGACTTCGCCGCATCAAAGCCGCAGAACCTGCCTCCTGAGTC
CTGTGCAAAAACACGGGCGCTGACAGAGCGTTATCATGAAGAGCGGGATG
CGTATGCATCCAATAAAGGTGGTACCACGGAAATGGAATTGCAGCCCTTT
TCGTCCTTTTCTAAAGGATGAAAAGGGCTTTTTTAATGAGGTCCCAGAGG
T-Box
P123_del_TF
GCCAGACCCGCTCTTCGTTAATGTATTAAAGCGATATCAATCTCATTCAC
GCATCTTTCTGCAACAGGAGGAAGTCGATATG GAT AAA
M D K
RBS
ProB
b
-35
-10
CACTTGACAGATACGCATGTTTTCTTGCATAATCACAATTAAAACTTATT
T-Box
ATGTAACGAAGCAATGAGTAAGGCCAGTAGATGGAAAACGGCGAAAGAGA
GTGAAGCTCACCGGCTGAAAGGCTTCACCGCCCTCTTGACCATTGAACCT
ACCTTACGAGCTGTTAGGCAAACCTAAACGCAAATCCGGCGTTATCGGAT
CAGAGCTGCACCTTTTCTGCTAGGCTTGACAGCCTTGAAAAGGTGAATGA
AGGTGGTACCACGGAAGATAGACCTTTCGTCCTTTGCGGATGTAAGGTCT
T-Box
proIWT2_pCF6_rv
TTTTTATTTTCATAAAATACAGAATGTTGAAATAAAAGATATGGGAGGAA
RBS
ACAAAAATG AAG AAG CTT
M K K L
ProI

Fig. S1 The predicted promoter and T-box regulatory regions of the *proBA* and *proI* anabolic L-proline biosynthetic genes from *Bacillus methanolicus* MGA3.

The DNA sequences of the upstream regions of (a) the *proBA* operon and (b) of the *proI* gene, as deduced from the genome sequence of *B. methanolicus* MGA3 [1], are shown. The putative SigA-dependent promoter elements (-35 and -10) and ribosome binding sites (RBS) are indicated. The predicted start codons of *proB* and *proI* are highlighted. The position of the DNA segment encoding the predicted T-box L-proline-responsive regulatory element [2, 3]**,** which was deleted in various plasmid constructs, is indicated and marked in red. The positions and the DNA sequences of the Primers used to delete the T-box sequence of the *proBA* (P124_del_T2F; P123_del-TF) and that used for the fusion of the *proI* gene (proIWT2pCF6-rv) to the *proBA* operon in the *proBA*-*proI* synthetic operon are indicates as well.

Figure S2 Predicted fold of the *proBA* and *proI* B-box mRNA riboswitches from *Bacillus methanolicus MGA3.*

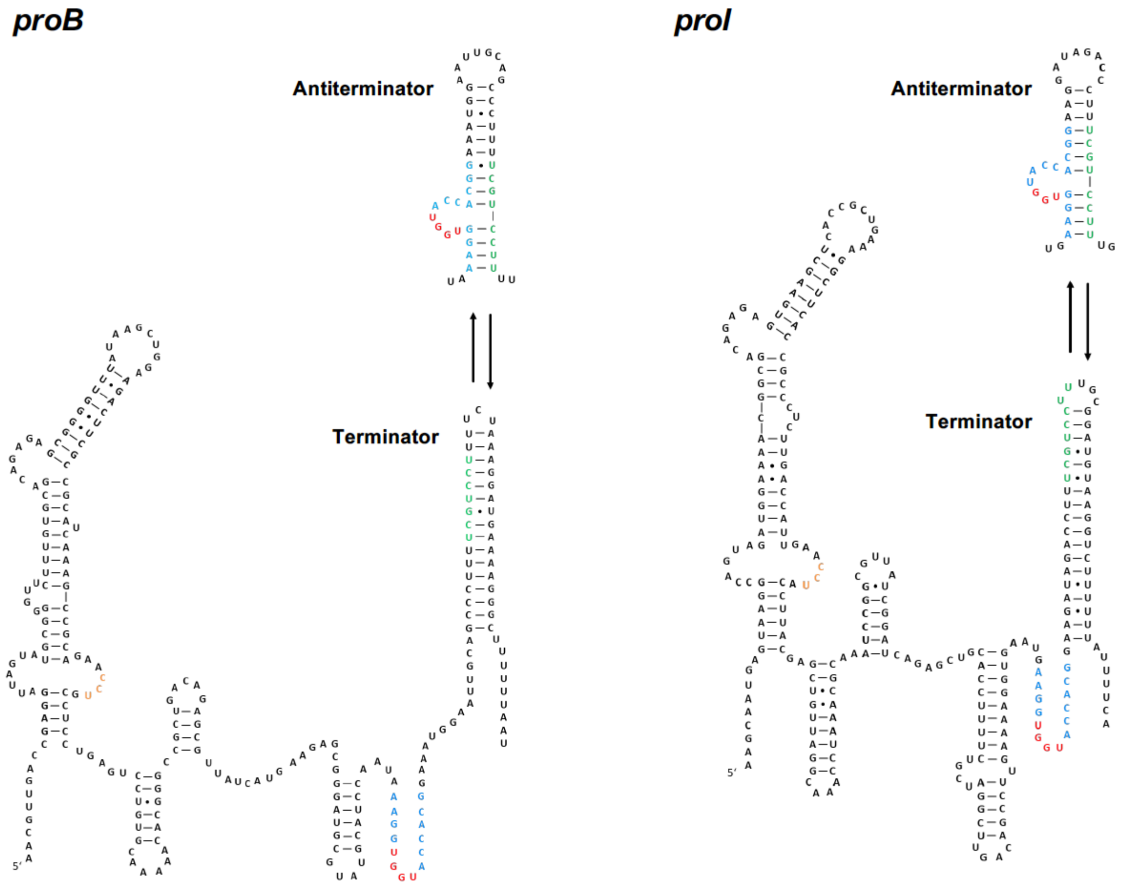

Fig. S2. Predicted mRNA secondary structures of the T-box elements in *proBA* and *proI*. The mRNA secondary structures were predicted using Mfold [4] and manually edited based on data from the corresponding *B. subtilis proBA* and *proI* genes [2]. The mutually exclusive terminator and anti-terminator structures of the T-box elements are shown. The predicted L-proline-specific specifier codon (CCU) and the anti-acceptor sequence (UGGU) are highlighted; these regions interact with uncharged tRNA^{Pro} to shift the T-box from the terminator to the anti-terminator conformation [3].

Fig. S3 Overview sketch on the construction of a synthetic proBA-proI anabolic L-proline biosynthetic operon from *Bacillus methanolicus* MGA3.

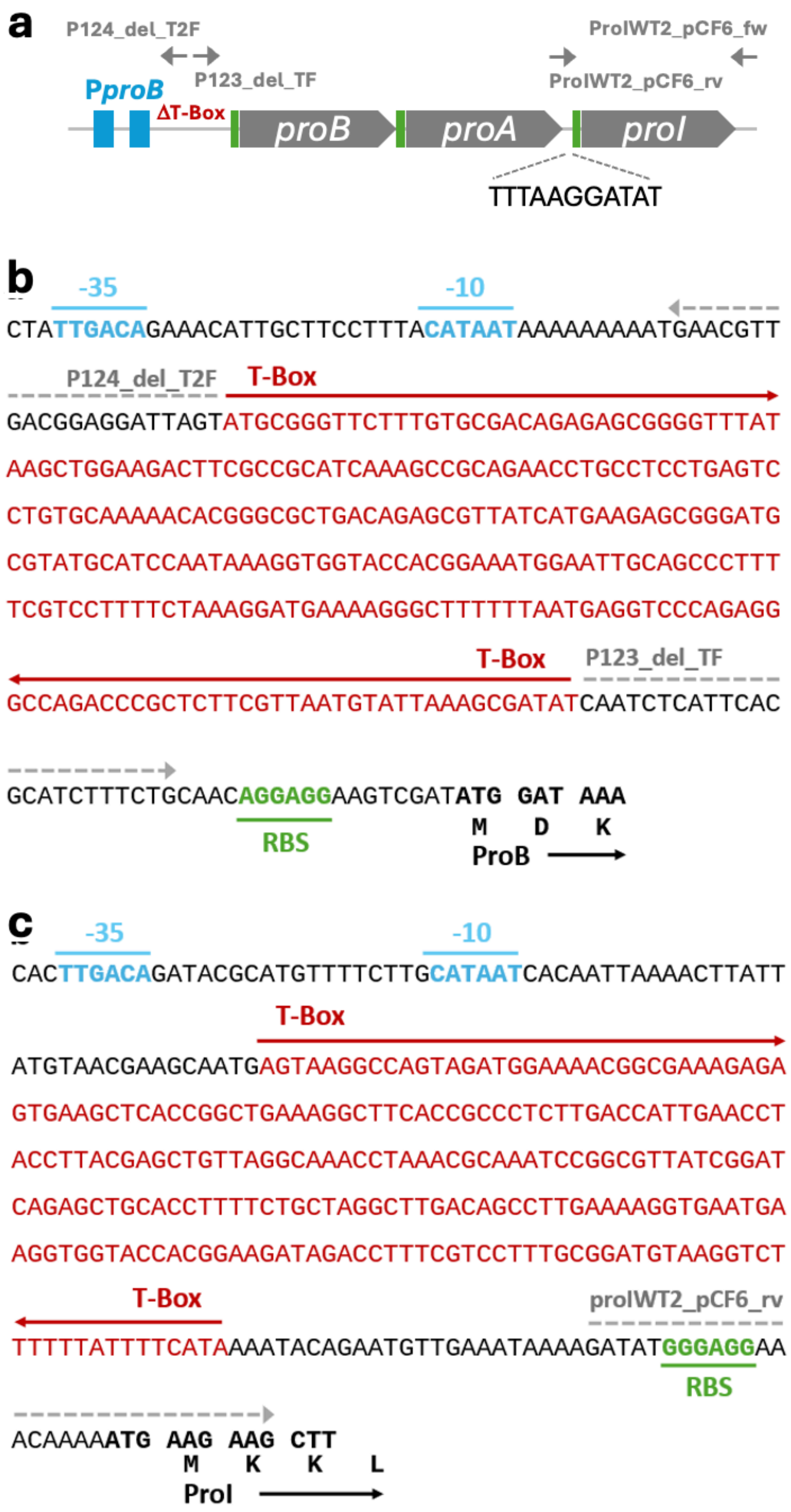

Fig. S3 Genetic design of a synthetic *proBA-proI* anabolic L-proline biosynthetic operon of *Bacillus methanolicus* MGA3.

Scheme of (a) the synthetic *proBA-proI* operon showing the position of the *proBA* promoter, the deleted region for the transcriptional T-box regulatory element, and the positions of the ribosome binding sites (RBS) (green boxes). The coding region of proI was fused to the 3'-end of the *proBA* operon using a short synthetic DNA segment. (b) The DNA sequences of the upstream regions of the *proBA* operon and (c) of the *proI* gene, as deduced from the genome sequence of *B. methanolicus* MGA3 [1], are shown. The putative SigA-dependent promoter elements (-35 and -10) and ribosome binding sites (RBS) are indicated. The predicted start codons of *proB* and *proI* are highlighted. The position of the DNA segment encoding the predicted T-box L-proline-responsive regulatory element [2, 3]**,** which was deleted in various plasmid constructs, is indicated and marked in red. The positions and the DNA sequences of the Primers used to delete the T-box sequence of the *proBA* (P124_del_T2F; P123_del-TF) and that used for the fusion of the *proI* gene (proIWT2pCF6-rv) to the *proBA* operon in the *proBA-proI* synthetic operon are indicates as well.

Figure S4 Use of various amino acids as sole carbon and nitrogen sources by *B. methanolicus* MGA3

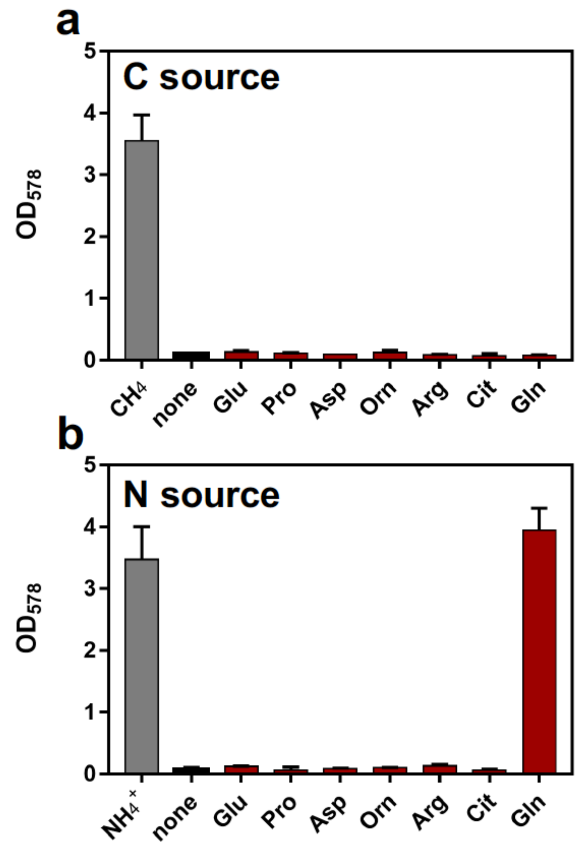

Fig. S4. *B. methanolicus* MGA3 is unable to utilize various amino acids, including L-proline, as a nutrient. Use of different amino acids as (a) sole carbon sources or (b) sole nitrogen sources was tested. (a) Sole carbon source: methanol (CH_4) in MVcM, normally used as the carbon and energy source, was replaced with the indicated amino acids. Amino acid concentrations were: 40 mM L-glutamate (Glu), 40 mM L-proline (Pro), 50 mM L-aspartate (Asp), 40 mM L-arginine (Arg), 40 mM L-glutamine (Gln), 40 mM L-ornithine (Orn), and 33 mM L-citrulline (Cit). (b) Sole nitrogen source: 16 mM $(NH_4)_2SO_4$ in MVcM was replaced with the indicated amino acids to provide nitrogen for growth. Amino acid concentrations were: 32 mM L-glutamate, 32 mM L-proline, 50 mM L-aspartate, 11 mM L-arginine, 16 mM L-glutamine, 40 mM L-ornithine, and 11 mM L-citrulline. - Data represent two biological replicates of cells grown at 50° C.

Figure S5 Influence of high salinity of recombinant *B. methanolicus* MGA3 L-proline biosynthetic cell factories

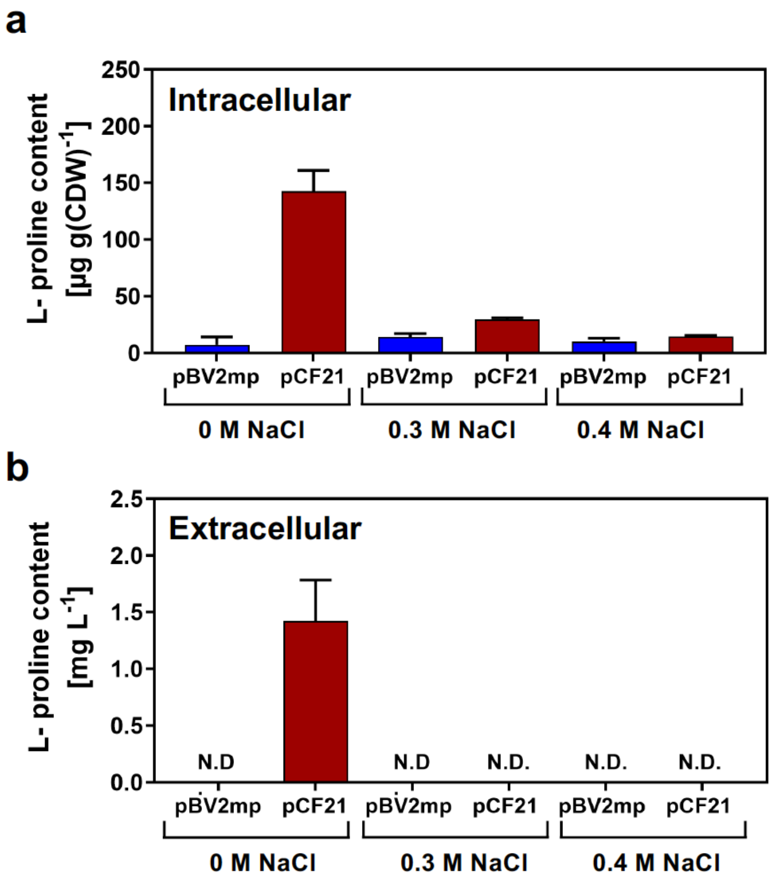

Fig. S4. Recombinant L-proline production under hyperosmotic conditions. *B. methanolicus* MGA3 strains carrying the plasmid pCF21-encoded L-proline biosynthetic *proB-proA-proI operon* under the transcriptional control of the mph promoter. Cells were cultivated in MVcM at different NaCl concentrations (0 mM, 0.3 M, and 0.4 M) at 50° C. Cells carrying the vector plasmid pBV2mp [5], used for construction of plasmid pCF21, were grown under the same conditions and served as controls. Cultures were harvested at an OD_{578} of 1.5. The intracellular (a) and extracellular (b) L-proline contents were quantified by HPLC. Data represent two independently grown cultures, with each HPLC measurement performed at least twice.

REFERENCES

1. Heggeset TM, Krog A, Balzer S, Wentzel A, Ellingsen TE, Brautaset T. Genome sequence of thermotolerant *Bacillus methanolicus*: features and regulation related to methylotrophy and production of L-lysine and L-glutamate from methanol. *Appl Environ Microbiol* 2012; 78:5170-5181.
2. Brill J, Hoffmann T, Putzer H, Bremer E. T-box-mediated control of the anabolic proline biosynthetic genes of *Bacillus subtilis*. *Microbiology* 2011; 157:977-987.
3. Gutierrez-Preciado A, Henkin TM, Grundy FJ, Yanofsky C, Merino E. Biochemical features and functional implications of the RNA-based T-box regulatory mechanism. *Microbiol Mol Biol Rev* 2009; 73:36-61.
4. Zuker M: Mfold web server for nucleic acid folding and hybridization prediction. *Nucleic Acids Res* 2003; 31:3406-4315.
5. Irla M, Heggeset TM, Naerdal I, Paul L, Haugen T, Le SB, Brautaset T, Wendisch VF. Genome-based genetic tool development for *Bacillus methanolicus:* theta- and rolling circle-replicating plasmids for inducible gene expression and application to methanol-based cadaverine production. *Front Microbiol* 2016, 7:1481.
