# Supplementary material for: Harnessing the thermotolerant methylotroph Bacillus methanolicus for methanol-based synthetic L-proline production
Source: Microb Cell Fact. 2026 May 23;25:129. doi: 10.1186/s12934-026-03032-8 (PMC13198045; doi:10.1186/s12934-026-03032-8)
Supplement: Supplementary file 3 — Supplementary Material 3. [file 12934_2026_3032_MOESM3_ESM.pdf]

TABLE S2 |Primers used to construct recombinant plasmids and site-directed mutagenesis

11_proHJAA_CF7fw:	CATAAATAGGAGGTAGGTACgaatctgccttcagcctcc
12_proHJAA_CF7_rv:	GAATTCGAGCTCATGggtgtctgacaaaccaggtg
10_proHJAAGi_fw:	AATCAAAGGGGGAAATGGGAATCTGCCTTCAGCCTC
P89_proBA_pHSG_fw:	CTATGACCATGATTACGAATTCCCCAATCTCATTCACGCATCTTTC
P90_proBA_pHSG_rv:	CATTTTTGTTTCCTCCCATATCCTTAAATTATTTGCGTATTTGACCATTC
P91_proI-pHSG_fw:	GTCAAATACGCAAATAATTTAAGGATATGGGAGGAAACAAAAATGAAG
P97_Q5_proB_E142R_fw:	CCAAATGTCAATCTTTCTACTGAAACC
P98_Q5_proB_E142R_rv:	CTATGACCATGATTACGAATTCCCCAATCTCATTCACGATCTTTC
P126_ProS:	ATAAATAGGAGGTAGGTACCATGGATAAAAAACGAATTGTCGTT
P127_ProS2:	GAATTCGAGCTCATGGTACCCATGGGGGACATAACGATTC
ProIWT2_pCF6_fw:	GCCAGTGAATTCGAGGCTCATGGTACCATGGGGGACATAACGATTC
proIWT2_pCF6_rv:	CAAATACGCAAATAATTTAAGGATATGGGAGGAAACAAAAATGAAGAAG
P124_del-TF	CAATCTCATTCACGCATCTTTCTG
P124_del_T2F	ACTAATCCTCCGTCAACGTTCA
