# Supplementary material for: Harnessing the thermotolerant methylotroph Bacillus methanolicus for methanol-based synthetic L-proline production
Source: Microb Cell Fact. 2026 May 23;25:129. doi: 10.1186/s12934-026-03032-8 (PMC13198045; doi:10.1186/s12934-026-03032-8)
Supplement: Supplementary file 4 — Supplementary Material 4. [file 12934_2026_3032_MOESM4_ESM.pdf]

TABLE S3 | Online measurements were conducted during L-proline production by *Bacillus methanolicus* MGA3 (pCF21) under fed-batch conditions. Cells were cultivated in a 5 L bioreactor at 50° C using a modified MVcMY medium over a period of 40.5 hours. Methanol was used as the carbon and energy source. The presented data represent combined results from two independent fermentation runs.

**Nr-1**	**Ferm. Time [h]**	**RPM**	**T [°C]**	**pH**	**pO2**	**OD 600**	**NH4 [mg L^{-1}]**	**PO_4 [mg L^{-1}]**	**Off-Line pH**	**MeOH [g L^{-1}]**	**DCW [g L^{-1}]**
**0**	0	1201	49,9	6,78	100	0,4	1266	4955	6,78	6	0
**1**	10,7	1201	50	6,5	59,3	8	1274	4762	6,48	6,9	3,6
**2**	17,2	1201	50,1	6,44	34,7	42,4	2295	2875	6,55	11,3	27,2
**3**	22	1201	50	6,46	29,1	56	4332	1623	6,46	14,6	44,5
**4**	26,4	1201	50	6,48	36,1	62,7	4354	2046	6,47	13,5	43,4
**5**	40,5	1201	50	6,49	99,5	65	7385	2017	6,62	-	39,7

**Nr-2**	**Ferm. Time [h]**	**RPM**	**T [°C]**	**pH**	**pO2**	**OD 600**	**NH4 [mg L^{-1}]**	**PO_4 [mg L^{-1}]**	**Off-Line pH**	**MeOH [g L^{-1}]**	**DCW [g L^{-1}]**
**0**	0	1199	49,9	6.8	100	0,4	1337	5155	6.83	6	0
**1**	10,7	1199	50	6.47	43.9	8.2	1268	4908	6.49	7.4	70
**2**	17,2	1199	50,1	6.43	35.7	37.9	2864	3145	6.55	9.6	307.5
**3**	22	1199	50	6.49	37.2	56.5	3302	1303	6.44	10.6	616.1
**4**	26,4	1199	50	6.49	31.1	65.1	5262	1819	6.46	15	950.8
**5**	40,5	1199	50	6.51	34.5	57.7	7251	1401	6.64	11.6	1145.3
